# Varifocal Meta‐Lens for Multifunctional Focusing and Imaging

**DOI:** 10.1002/advs.202514015

**Published:** 2025-09-22

**Authors:** Rong Lin, Jin Yao, Chen Chen, Jiajun Wu, Junxiao Zhou, Zhihui Wang, Din Ping Tsai

**Affiliations:** ^1^ Department of Electrical Engineering City University of Hong Kong Kowloon Hong Kong 999077 China; ^2^ National Laboratory of Solid State Microstructures Key Laboratory of Intelligent Optical Sensing and Manipulations, Jiangsu Key Laboratory of Artificial Functional Materials, College of Engineering and Applied Sciences Nanjing University Nanjing 210093 China; ^3^ Centre for Biosystems Neuroscience and Nanotechnology City University of Hong Kong Kowloon Hong Kong SAR 999077 China; ^4^ The State Key Laboratory of Terahertz and Millimeter Waves City University of Hong Kong Kowloon Hong Kong SAR 999077 China; ^5^ Department of Physics City University of Hong Kong Kowloon Hong Kong 999077 China

**Keywords:** multifunctional, non‐diffractive, tunable, varifocal meta‐lens

## Abstract

Integrating multifunctionality and tunability in beam control is crucial for advanced applications such as adaptive imaging and optical microscopy. However, achieving both capabilities simultaneously in a compact optical system remains challenging, as conventional optics are bulky and typical tunable metasurfaces offer only limited operational modes. Here, polarization‐independent varifocal meta‐lens is presented, composed of two cascaded metasurfaces, enabling mode switching between a non‐diffracting abrupt autofocusing (AAF) beam and a diffraction‐limited focusing beam. The mode transition is governed by the dual interpretation of the superimposed phase profile in real space and the spatial frequency domain. A Moiré‐based tuning mechanism enables continuous focal length modulation via in‐plane rotation. The proposed varifocal meta‐lens achieves a focal length tuning range of 10.3 mm in AAF mode (163% tuning ratio) and 29.3 mm in standard focusing mode (345% tuning ratio). The AAF beam maintains a nearly constant spot size throughout its range, while the standard focusing mode supports brightfield imaging with a relative zoom magnification from 1× to 1.95×. This compact platform offers a promising solution for tunable and integrated optical systems.

## Graphical abstract

A varifocal meta‐lens enables dynamic switching between conventional focusing and non‐diffracting autofocusing beams, relying on a phase design with dual interpretations in both real space and the spatial frequency domain. By integrating a Moiré‐based tuning mechanism, the system achieves continuous focal length control in both modes. This compact, polarization‐independent platform offers versatile focusing and imaging for portable and integrated photonic applications.

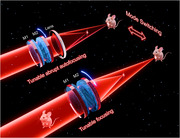

## 1 Introduction

The rapid development of portable imaging and chip‐scale photonics is accelerating the demand for compact, multifunctional optical systems that can deliver high performance in miniaturized platforms.^[^ ^1^, ^2^ ^]^ Conventional optical imaging systems, such as microscopes, rely on mechanically actuated *z*‐stages and switchable objective lenses to achieve depth‐resolved and variable‐magnification imaging.^[^ ^3^ ^]^ While effective, these systems are inherently bulky and mechanically complex, hindering their integration into miniaturized or portable applications. Various tunable optical components^[^ ^4^, ^5^, ^6^, ^7^ ^]^—such as spatial light modulators, liquid crystal lenses, and elastomeric lenses—have been explored to address this challenge. However, these devices often involve trade‐offs in resolution, tuning range, response speed, and long‐term stability, restricting their applicability in high‐performance, compact optical systems.

Metasurfaces,^[^ ^8^, ^9^, ^10^, ^11^ ^]^ composed of subwavelength nanostructures, offer a promising alternative by enabling precise control over the phase, amplitude, and polarization of light.^[^ ^12^, ^13^, ^14^ ^]^ Recent efforts have demonstrated dynamical control through Moiré phase modulation,^[^ ^15^, ^16^, ^17^ ^]^ Alvarez designs,^[^ ^18^, ^19^, ^20^ ^]^ interlayer spacing variation,^[^ ^21^ ^]^ incident states switching,^[^ ^22^, ^23^, ^24^ ^]^ and active materials responsive to mechanical,^[^ ^25^, ^26^, ^27^ ^]^ optical,^[^ ^28^ ^]^ or electrical stimuli.^[^ ^29^, ^30^, ^31^, ^32^, ^33^ ^]^ These advances have enabled applications in wavefront shaping, brightfield imaging, and quantitative phase measurement.^[^ ^34^, ^35^ ^]^ Nevertheless, most tunable metasurfaces to date are limited to modulating a single‐mode, diffraction‐limited focusing beam, restricting their use in emerging applications that demand more complex optical fields. In many emerging photonic applications—such as optical trapping, laser microsurgery, and deep‐tissue imaging—non‐diffracting beam profiles^[^ ^36^, ^37^, ^38^, ^39^ ^]^ are prefered due to their extended focal length and propagation stability. Especially AAF beams, which combine the self‐acceleration of Airy beams with the tightly focused profiles of Bessel beams,^[^ ^40^, ^41^, ^42^ ^]^ have obtained significant interest. Although passive metasurfaces implementations of AAF beam have been demonstrated for fluorescence microscope,^[^ ^43^ ^]^ underwater wireless optical communication,^[^ ^44^ ^]^ and transthoracic ultrasound therapy,^[^ ^45^ ^]^ their static nature excludes dynamic adaptation to varying imaging or treatment conditions.

Here, we present a varifocal meta‐lens comprising two cascaded metasurfaces, capable of dynamically switching between and continuously tuning two beam modes: a diffraction‐limited focusing beam and a non‐diffracting AAF beam. The beam mode transition is governed by the dual interpretation of the superimposed phase profile in real space and the spatial frequency domain. A Moiré‐based tuning mechanism enables continuous focal length adjustment for both modes via the relative in‐plane rotation between the two metasurfaces. Experimental results show that the AAF beam exhibits a tunable focal length from 6.3 to 16.6 mm, corresponding to a tuning ratio of 163%, while maintaining a constant full‐width at half‐maximum (FWHM) of the focal spot, confirming its non‐diffracting characteristic. The standard focusing mode achieves a broader tuning range from 8.5 to 37.8 mm (345% tuning ratio), enabling variable‐magnification brightfield imaging from 1× to 1.95×. This work provides a compact and polarization‐insensitive solution for multifunctional beam shaping and varifocal imaging. The ability to dynamically access both diffraction‐limited and non‐diffracting beams in a single meta‐system offers an option for integrated optical systems in portable microscopy and adaptive imaging.

## 2 Results

### 2.1 Design Principle of the Proposed Varifocal Meta‐Lens

**Figure 1:**
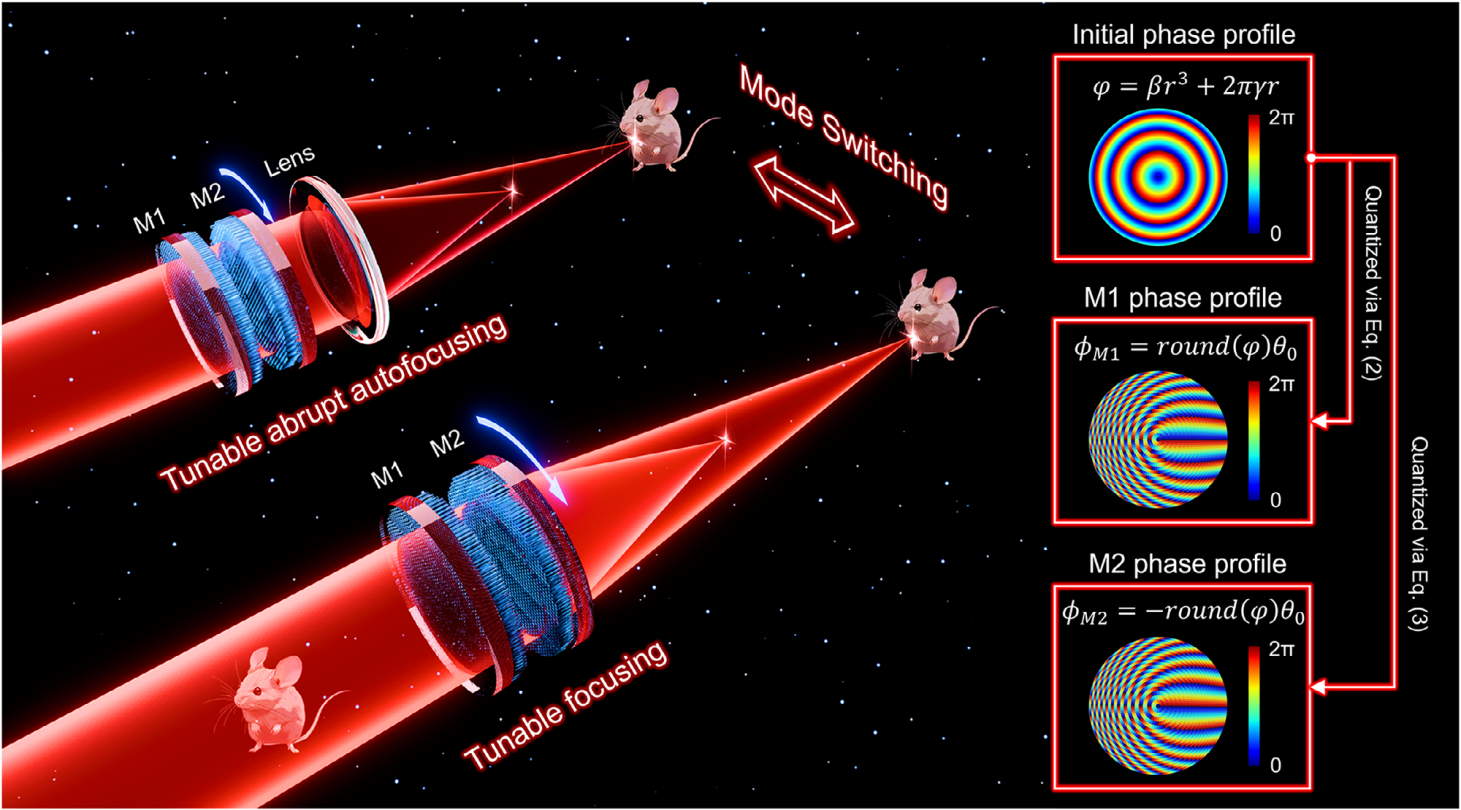
Schematic illustration of the varifocal meta‐lens for multifunctional beam shaping and imaging. The beam mode can be dynamically switched by selecting the appropriate interpretation domain, either real space or spatial frequency, which is controlled by inserting or removing a Fourier lens. The non‐diffracting AAF beam is suitable for applications such as laser microsurgery, while the standard focusing beam enables brightfield imaging. Continuous focal length tuning for both modes is realized by adjusting the relative in‐plane rotation between metasurface 1 (M1) and metasurface 2 (M2). The inset on the right shows the initial superimposed phase distribution, along with the ideal decomposed phase profiles for M1 and M2.

The initial superimposed phase distribution is shown at the top right corner of **Figure** **1** according to the following equation:^[^ ^43^, ^46^ ^]^

(1) $$\varphi =\beta r^{3}+2\pi \gamma r$$

where *β* and *γ* are two adjustable parameters related to beam properties, including focal length and shape, and *r* denotes the position in polar coordinates. The generation of two distinct beam modes lies in the dual interpretation of the superimposed phase distribution in the real space and the spatial frequency domain. In addition to realizing the standard focusing function in the real space directly, such a phase distribution corresponds to the Fourier transform of the AAF beam (a detailed derivation process is illustrated in Section 1 in the Supporting Information).

On the basis of the Moiré principle,^[^ ^47^, ^48^ ^]^ two different phase distributions (see M1 and M2 on the right side of Figure 1) can be formed as shown in Equations (2) and (3):

(2) $$\phi_{M1}\left(r,\theta_{0}\right)=round\left(\varphi \right)\cdot \theta_{0}$$

(3) $$\phi_{M2}\;\left(r,\theta_{0}\right)=-round\left(\varphi \right)\cdot \left(\theta_{0}-\theta \right)$$

here, *θ*_0_ represents the reference polar angle of each point in the aperture, defined as $\theta_{0}=tan^{-1}\;\left(\frac{y}{x}\right)$, and θ represents the rotation angle. Note that in the case shown in Figure 1, the rotation angle is set to *θ* = 0°. The function *round*(·) performs nearest‐integer rounding of its argument, which serves as an effective strategy to eliminate discontinuities that may arise when the polar angle approaches π. Changing the relative rotation angle *θ* between the two phase distributions will not disrupt the form of the superimposed phase distribution, only influence the value of adjustable parameters as described in equation (4):

(4)

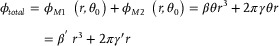




Thus, the standard focusing beam and AAF beam can be generated, and the focal length is manipulated by the rotation angle *θ*. The schematic diagram is shown in Figure 1, such a varifocal meta‐lens is composed of two metasurfaces M1 and M2, and beam mode switching happens on whether to insert a lens after M2. In detail, inserting a lens leads the superimposed phase distribution to complete a Fourier transform for generating an AAF beam, removing the lens will generate a standard focusing beam. Furthermore, changing the relative rotation angle *θ* will manipulate the focal length of both generated beams to satisfy different application scenarios.

**Figure 2:**
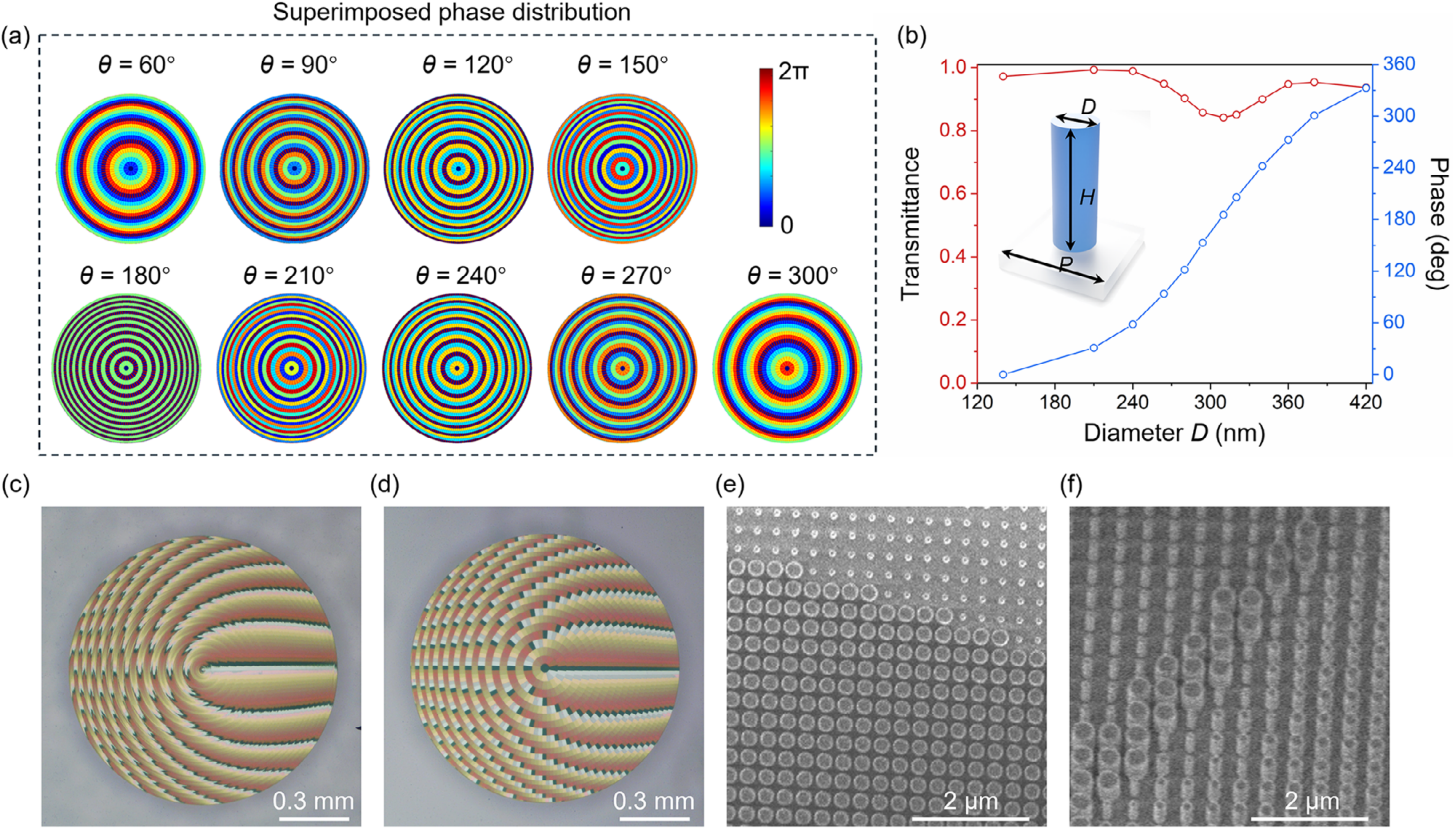
Design, phase modulation, and structural characterization of the varifocal meta‐lens. a) Superimposed phase distributions at different relative rotation angles *θ*. b) Transmittance and propagation phase of the unit cell as functions of diameter *D* at an incident wavelength of 1310 nm, the period *P* = 520 nm, and the height *H* = 800 nm. Optical microscopy images of c) M1 and d) M2. Scanning electron microscopy (SEM) images showing sub‐regions of e) M1 and f) M2. The diameter of the whole meta‐lens is 1.15 mm.

**Figure** **2**a shows the superimposed phase distribution at different relative rotation angles *θ*. Here, the operational wavelength is set at 1310 nm. The inserted image in Figure 2b shows the proposed unit cell consisting of a silicon nanopillar on a K9 glass substrate; the refractive index of silicon is depicted in Figure S2 (Supporting Information). To satisfy the phase distribution of M1 and M2, the silicon nanopillars with a suitable diameter *D* are chosen to realize 2π full phase coverage as depicted in Figure 2b, which also presents the corresponding transmittance under the linear polarization state. It should be noted that the phase distribution of M1 and M2 shown in Figure 1 is an ideal one. However, in a practical situation, a finite air gap must be introduced between the two cascaded metasurfaces to prevent physical damage to the fabricated nanostructures. This gap allows the output field from the first metasurface (M1) to propagate freely before reaching the second (M2), resulting in a deviation of the actual superimposed phase distribution from the predesigned target. This mismatch degrades the quality of the generated optical beam, particularly in applications requiring high phase fidelity. To prevent physical contact and potential damage between the two metasurfaces during rotation and alignment, we introduce a 1 mm spacing between them. The phase profile of M1 was optimized to minimize the resulting phase mismatch due to this finite spacing. The detailed optimization process and corresponding evaluation for the optimization strategy is provided in Section 3 of the Supporting Information. Figure 2c,d show the optical microscopy images of the two fabricated metasurfaces, and the SEM images corresponding to sub‐regions of M1 and M2 are shown in Figure 2e,f, respectively. More SEM images are presented in Figure S8 (Supporting Information). The detailed fabrication process is referred to in Figure S9 (Supporting Information) and described in Methods.

### 2.2 Experimental Results of AAF Beams' Propagation Properties

**Figure 3:**
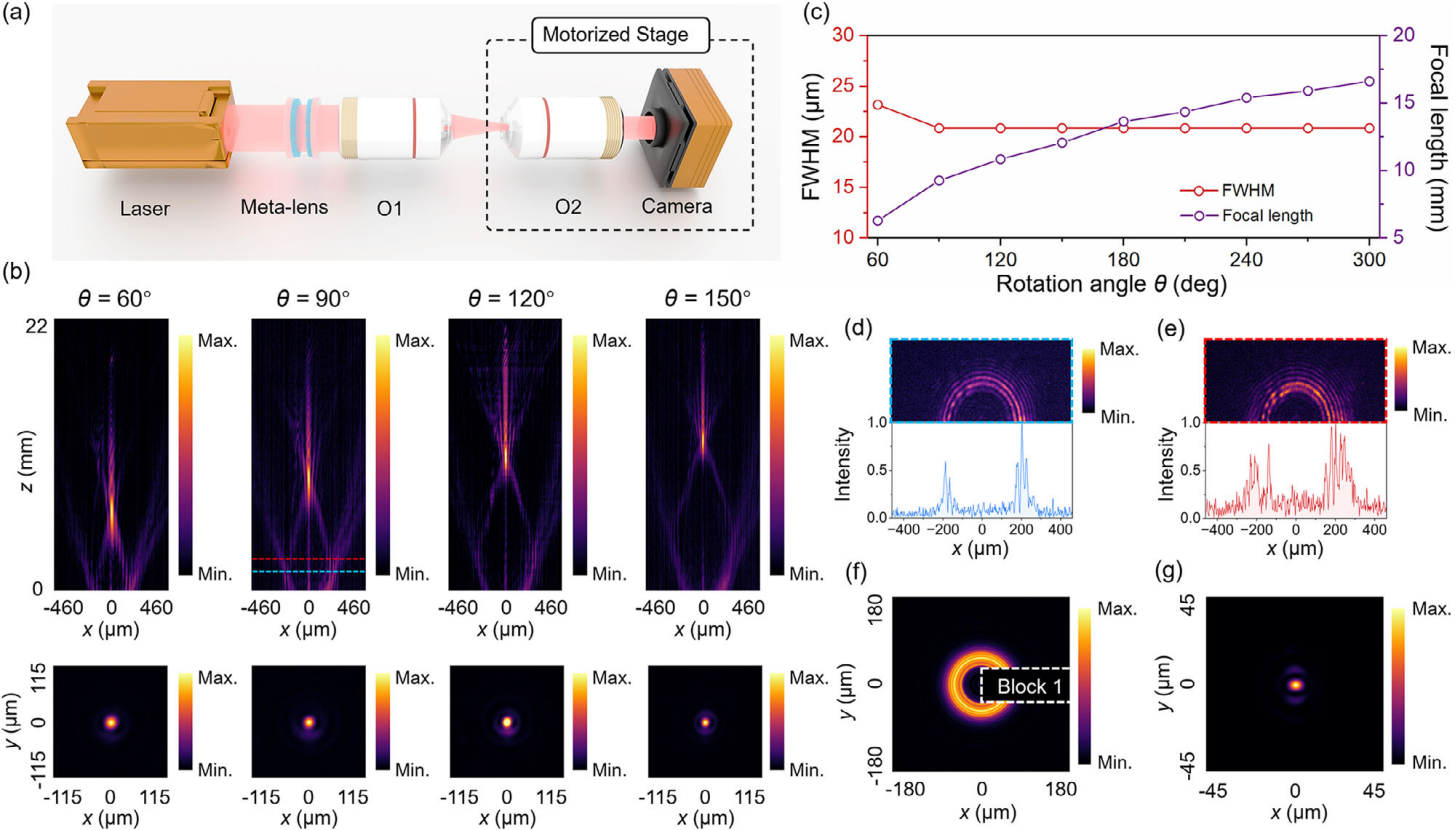
Tunable abrupt autofocusing generated by the varifocal meta‐lens. a) Schematic of the experimental setup. b) Experimental *x‐z* intensity cross‐sections and corresponding *x‐y* intensity distributions at the focal plane under the relative rotation angle *θ* = 60°, 90°, 120°, and 150°. c) Measured FWHM of the focal spot and effective focal length as functions of relative rotation angle. d) Half‐plane *x‐y* intensity distribution (blue dashed line position) and its corresponding lateral intensity distribution along the *x*‐axis. e) Half‐plane *x‐y* intensity distribution (red dashed line position) and its corresponding lateral intensity distribution along the *x*‐axis. f) Numerical *x‐y* intensity distribution at *θ* = 90° with obstruction. g) Corresponding focal plane *x‐y* intensity distributions.

To validate the tunable non‐diffracting behavior of the proposed varifocal meta‐lens, we first characterized the generation of AAF beams under different relative rotation angles. Numerical analysis of the intensity distributions and related metrics is provided in Figures S10,S11 (Supporting Information). To balance computational efficiency with accuracy, the Fourier lens used in the simulation was set to be three times larger in aperture than the meta‐lens, ensuring complete collection of the transmitted light. The experimental setup is depicted in **Figure** **3**a. In this configuration, an objective O1 serves as a Fourier‐transforming tool to facilitate the formation of AAF beams from the designed phase profile. Full characterization details are provided in the Methods section.

Figure 3b shows the measured intensity distribution in the *xz*‐plane, as well as the corresponding focal plane intensity profiles, at selected relative rotation angles *θ* = 60°, 90°, 120°, and 150° under 1310 nm illumination. Intensity distributions at other relative rotation angles are presented in Figure S12 (Supporting Information). These exhibit a distinct transition from concentric circle distribution to a sharply Bessel‐like spot, consistent with the defining features of AAF beams.

Since the exact back focal plane of the objective is not explicitly known, we define the axial origin (*z* = 0 mm) as the plane where the concentric circle pattern becomes clearly resolved in the experiment. Based on this definition, the effective focal length is found to increase from 6.3 to 16.6 mm as *θ* increases, while maintaining a nearly constant FWHM of ∼21 µm, as summarized in Figure 3c. These variation trends are in strong agreement with theoretical predictions. A weak axial intensity observed near *x* = 0 in the *xz*‐plane intensity distribution is attributed to residual unmodulated light. This component, directly focused by the objective, does not participate in the beam‐shaping process. After excluding this contribution, the AAF beam exhibits a well‐defined hollow‐ring profile during propagation, as illustrated in Figure 3d,e. The upper panels display transverse *xy*‐plane intensity slices at two positions (indicated by dashed lines in Figure 3b), and the bottom panels reveal the corresponding lateral intensity profiles across the center. Minor asymmetries in intensity are likely due to fabrication imperfections, interlayer misalignment, and non‐ideal collimation in the optical path, all of which can be further optimized in future iterations.

To assess the self‐healing property of the generated AAF beam, we numerically introduced an opaque obstacle (Block 1) at the initial plane to partially obstruct the incident field. As shown in Figure 3f, even with blockage at *θ* = 90°, the resulting focal spot (Figure 3g) remains largely unaffected, demonstrating its inherent self‐healing capability. Additional numerical analysis across other rotation angles, shown in Figure S13 (Supporting Information), further reveals its robustness.

### 2.3 Experimental Results of Standard Focusing Beams

**Figure 4:**
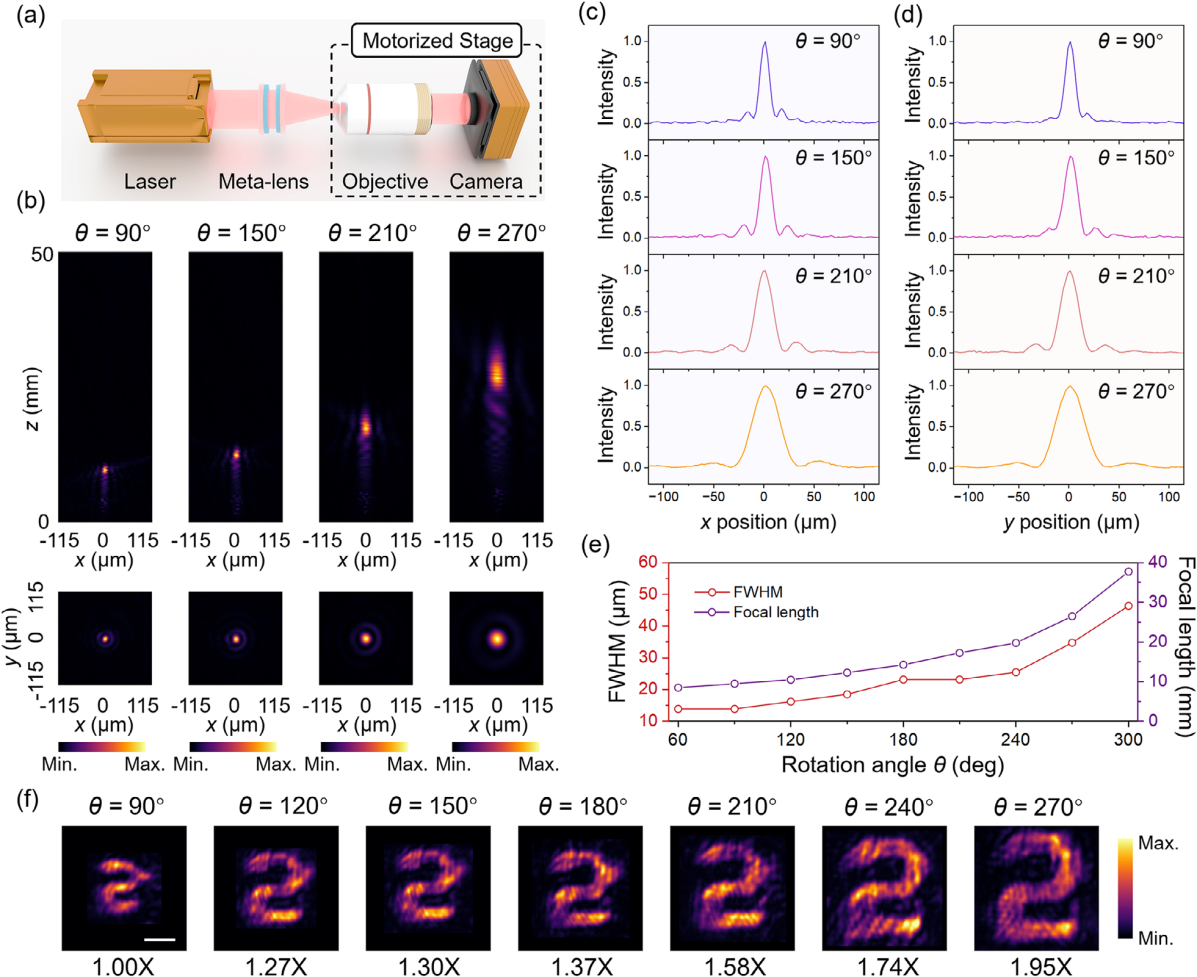
Tunable focusing generated by the varifocal meta‐lens. a) Schematic of the experimental setup. b) Experimental *x‐z* intensity cross‐sections (top panels) and corresponding focal plane *x–y* intensity maps (bottom panels) at different relative rotation angles. Transverse intensity profiles through focal spot centers under varying *θ* along the c) *x*‐axis and d) *y*‐axis. e) Measured focal length and FWHM of the focal spot as functions of rotation angle. f) Imaging results at different relative rotation angles.

To evaluate the standard focusing performance of the proposed varifocal meta‐lens, the Fourier‐transforming objective was removed, allowing the transmitted light to be directly collected, as depicted in **Figure** **4**a. Representative intensity distributions in both the *x‐z* propagation plane and the *x‐y* focal plane are shown in Figure 4b for relative rotation angle *θ* = 90°, 150°, 210°, and 270°, with additional experimental results provided in Figure S14 (Supporting Information). Detailed evaluation metrics, such as focusing efficiency, are explained in Section S9 (Supporting Information). As *θ* increases, a clear elongation of the focal length and broadening of the focal spot are observed, as quantified by the transverse intensity profiles across the center of focal spots along the *x*‐ and *y*‐axes (Figure 4c,d). Different from the AAF beam mode, the standard focusing mode exhibits a significantly extended focal tuning range, reaching from 8.5 to 37.8 mm as *θ* varies from 60° to 300° presented in Figure 4e. Despite this wide tuning range, the focal spot maintains a sub‐diffraction‐limited FWHM across all situations. These trends are in excellent agreement with numerical simulations (Figure S16, Supporting Information), confirming the consistency and predictability of the design. The proposed varifocal meta‐lens enables brightfield imaging with tunable magnification, making it suitable for zoom imaging applications. A digit “2” is chosen as the target object, as shown in Figure 4f, clear and well‐resolved images were obtained across seven distinct rotation states. Taking the image at *θ* = 60° as the reference, the relative optical magnification was found to vary continuously from 1× to 1.95× as the rotation angle increased.

## 3 Discussions and conclusion

In conclusion, the proposed dual‐mode varifocal meta‐lens introduces a compact platform for multifunctional beam generation, capable of dynamically switching between a standard diffracted‐limited focusing beam and a non‐diffracting AAF beam. This mode transition is governed by a dual‐space interpretation of a superimposed phase profile, enabling control over beam type and focal length within a single integrated system. In AAF mode, the beam maintains a nearly constant focal spot size over a continuously tunable focal length range from 6.3 to 16.6 mm, which is particularly valuable in applications such as optical trapping and laser microsurgery. Conversely, the standard focusing mode supports brightfield imaging over a broader range (8.5 – 37.8 mm), enabling zoom imaging with a relative magnification from 1× to 1.95×. To quantitatively assess the device's tunability, we define the tuning ratio as (*f_max_* − *f_min_*)/*f_min_*, which corresponds to 163% in AAF mode and 345% in the standard mode—both exceeding those of the tunable meta‐lens as summarized in Table S2 (Supporting Information). Compared to prior Moiré‐based metasurfaces, which have been largely limited to tuning diffraction‐limited focal spots, our device extends tunability to a qualitatively different beam class. By generating non‐diffractive AAF beams with Airy‐Bessel characteristics, it enables longitudinally extended, spatially confined energy delivery within a continuously adjustable focus range. This capability is particularly relevant for biomedical applications requiring precise and dynamic beam control. Such dual‐mode functionality is particularly valuable in practical scenarios that require switching between localized energy delivery and extended‐depth irradiation. For example, in laser microsurgery or phototherapy, the ability to switch between a tightly focused beam for cutting and an AAF beam for depth‐selective energy delivery can offer greater precision and safety. Similar benefits extend to volumetric imaging, optical trapping, and high‐resolution material processing, where beam mode adaptability can significantly enhance system performance across diverse tasks. Moreover, our system supports dual‐mode operation without requiring material reconfiguration, spatial multiplexing, or polarization control.

It should be noted that the phase profile used in our design, although deviating from an ideal quadratic lens, is intentionally structured to combine a cubic and linear radial term. This hybrid profile enables dual‐domain operation by focusing directly in real space and generating an AAF beam in the Fourier domain. While this approximation introduces minor aberrations in the standard focusing mode, it represents a deliberate trade‐off that prioritizes functionality. Moreover, the known deviation allows for computational correction using post‐processing or learning‐based techniques, which can further improve imaging fidelity where needed. The distinct responses of the metasurface in real space and Fourier space offer a strategy for information‐rich metasurface design. Our meta‐lens uniquely supports dual‐mode beam shaping, continuous tuning, polarization insensitivity, and imaging validation. Additionally, this design mechanism can be extended across different electromagnetic frequency regimes and accompanied with an integrated‐resonant unit (IRU)^[^ ^49^, ^50^, ^51^ ^]^ to broaden the operational bandwidth. Although a conventional objective lens was used in our experimental demonstration to perform the Fourier transform required for dual‐mode operation, it is not essential to the proposed concept. This function can be fulfilled by any optical element capable of Fourier transformation, such as a plano‐convex lens. As demonstrated in Section S6 (Supporting Information), a simple lens with a larger aperture can achieve the desired autofocusing behavior. Looking ahead, this component can be further miniaturized or replaced with a flat meta‐lens, facilitating full system integration. Thus, the proposed design remains compatible with compact and multifunctional optical architectures, without fundamentally relying on bulky optical elements. These results not only expand the capabilities of varifocal meta‐lens but also pave the way for compact, tunable, and application‐specific optical systems in fields ranging from biomedical imaging to adaptive optical sensing.

## 4 Experimental Section

### Simulation

Electromagnetic responses of the meta‐atoms were numerically simulated using the finite element method (FEM) implemented in COMSOL Multiphysics. The simulations were performed under normally incident, *x*‐polarized plane wave illumination. Periodic boundary conditions were applied along the *x*‐ and *y*‐directions, while perfectly matched layers (PMLs) were placed at the top and bottom boundaries. The refractive index of Si is provided in Figure S2 (Supporting Information), and the K9 glass substrate was modeled with a constant refractive index of 1.51. Far‐field intensity distributions were computed by the Rayleigh‐Sommerfeld diffraction algorithm.

### Fabrication

The fabrication procedure is illustrated in Figure S9 (Supporting Information). An 800 nm‐thick Si layer was deposited on a K9 glass substrate first. Subsequently, an 80 nm PMMA resist layer was spin‐coated and patterned via electron beam lithography using an Elionix F125 system, followed by standard development. A 40 nm chromium (Cr) layer, serving as a hard mask, was then deposited using an electron beam evaporator (ULVAC ei‐501z), followed by the lift‐off process. Next, the Si layer was etched through inductively coupled plasma etching (Leuven, ICP), and the residual Cr mark was removed by immersing the sample in chromium etchant.

### Measurement

To acquire three‐dimensional light‐field data, a supercontinuum laser with an acousto‐optic tunable filter (AOTF) was used to generate single‐wavelength illumination. Two metasurfaces were individually mounted on motorized rotation stages, which were in turn installed on high‐precision three‐axis translation stages. To characterize the generated AAF beam mode, an objective (O1) was employed to perform a Fourier transform, then the output light field was captured by a second objective (O2) and a near‐infrared CCD camera mounted on a motorized translation stage (LBTEK). In contrast, for testing the standard focusing mode, O1 was removed, allowing the output from the metasurfaces to be directly collected by O2 and the CCD camera.

## Conflict of Interest

The authors declare no conflict of interest.

## Supporting information



Supporting Information

## Data Availability

The data that support the findings of this study are available from the corresponding author upon reasonable request.
