# Supplementary material for: Varifocal Meta‐Lens for Multifunctional Focusing and Imaging
Source: Adv Sci (Weinh). 2025 Sep 22;12(45):e14015. doi: 10.1002/advs.202514015 (PMC12677681; doi:10.1002/advs.202514015)
Supplement: Supplementary file 1 — Supporting Information [file ADVS-12-e14015-s001.docx]

Supporting Information

Varifocal meta-lens for multifunctional focusing and imaging

*Rong Lin^†^, Jin Yao^†,*^, Chen Chen, Jiajun Wu,* *Junxiao Zhou, Zhihui Wang, and Din Ping Tsai**^*^*

R. Lin, J. Yao, C. Chen, Z. Wang, J. Zhou, D. P. Tsai

Department of Electrical Engineering

City University of Hong Kong, Kowloon, Hong Kong 999077, China

C. Chen, J. Zhou
National Laboratory of Solid State Microstructures, Key Laboratory of Intelligent Optical Sensing and Manipulations, Jiangsu Key Laboratory of Artificial Functional Materials, College of Engineering and Applied Sciences

Nanjing University, Nanjing, 210093, China

D. P. Tsai

Centre for Biosystems, Neuroscience and Nanotechnology

City University of Hong Kong, Kowloon, Hong Kong SAR 999077, China

D. P. Tsai

The State Key Laboratory of Terahertz and Millimeter Waves

City University of Hong Kong, Kowloon, Hong Kong SAR 999077, China

D. P. Tsai

Department of Physics

City University of Hong Kong, Kowloon, Hong Kong 999077, China

*^*^*Corresponding Authors**,** E-mails: jinyao@cityu.edu.hk; mkchen@cityu.edu.hk; dptsai@cityu.edu.hk

*^†^*These authors contributed equally to this work.

**Section 1: Derivation process for generating AAF beams through metasurfaces**

The optical field of the generated AAF beam can be mathematically described by a modulated Airy-like function, as shown in Eq. (S1):

$E_{0}=Ai\left( \frac{r_{0}-r}{w} \right)e^{\frac{m(r_{0}-r)}{w}}$ (S1)

Here $Ai$ denotes the Airy function, $r$ is the radius coordinate, $r_{0}$ corresponds to the radius of the main intensity lobe, $w$ is a scaling parameter, and $m$ represents an exponential decay factor. During free-space propagation, the beam gets autofocusing, reaching its focal spot at a distance $f_{A}$, given by^[1]^

$f_{A}= \frac{4\pi}{\lambda}\sqrt{w^{3}{(r}_{0}+w)}$ (S2)

To realize such a beam profile experimentally, we designed a computer-generated phase mask located at the Fourier plane. This design is based on a cubic-phase-modulated conic phase function, which encodes the desired propagation dynamics into the spatial frequency domain. The phase modulation applied to the mask follows the form^[2]^:

$\varphi=\beta r^{3}+2\pi\gamma r$ (S3)

where *β* and *γ* are adjustable parameters related to the spatial phase profile distribution. Upon illumination and Fourier transformation through a lens, the encoded phase profile gives rise to a spatially accelerating beam exhibiting abrupt autofocusing characteristics^[3]^. In our implementation, the design parameters were selected as *β* = ${5\times10}^{10}$, and *γ* = 5000 to match the desired beam trajectory and focal position.

It should be noted that the superimposed phase distribution is given by:

$\phi_{total}=\phi_{M1}\left( r,\theta_{0} \right) + \phi_{M2}\left( r,\theta_{0}-\theta\right)={\beta\theta r^{3}+2\pi\gamma\theta r=\beta}^{'}r^{3}+2\pi\gamma^{'}r$ (S4)

Here the coefficients β' and γ' depend on the initial design parameters β and γ, as well as rotation angle *θ*. While their specific values vary with design, the phase profile fundamentally combines a nonlinear (cubic) term and a linear radial term.

When the phase $\phi_{total}$ is directly imprinted onto a collimated input wavefront, the resulting beam is:

$E\left( r \right)=\exp\left[ i\phi_{total}\left( r \right) \right]$ (S5)

The local spatial frequency (first derivative of phase) is:

${\phi^{'}}_{total}\left( r \right)=2\pi\gamma^{'}+3\beta^{'}r^{2}$ (S6)

The expression shows that wavefronts corresponding to larger radii accumulate phase more rapidly and bend inward, leading to a constructive collapse toward the propagation axis. This results in a focusing effect in free space. Thus, even without any Fourier-transforming optics, the beam focuses spontaneously at a certain propagation distance due to the radial chirp-induced inward collapsing of wavefronts.

The same phase profile can also produce an AAF beam when subjected to a Fourier transform. According to the analytical derivation in Ref. [3], an AAF beam arises from a spectrum of the form:

$U\left( k \right)\sim J_{0}(kr_{0}+\frac{k^{3}}{3})$ (S7)

This expression implies that the far-field amplitude of the AAF beam is proportional to a beam that we generate in the free space. Therefore, if we encode this using $\phi_{total}$ and apply a Fourier lens, the output can perform an abrupt autofocusing function. Figure S1 describes the process of the dual-mode beam generation.


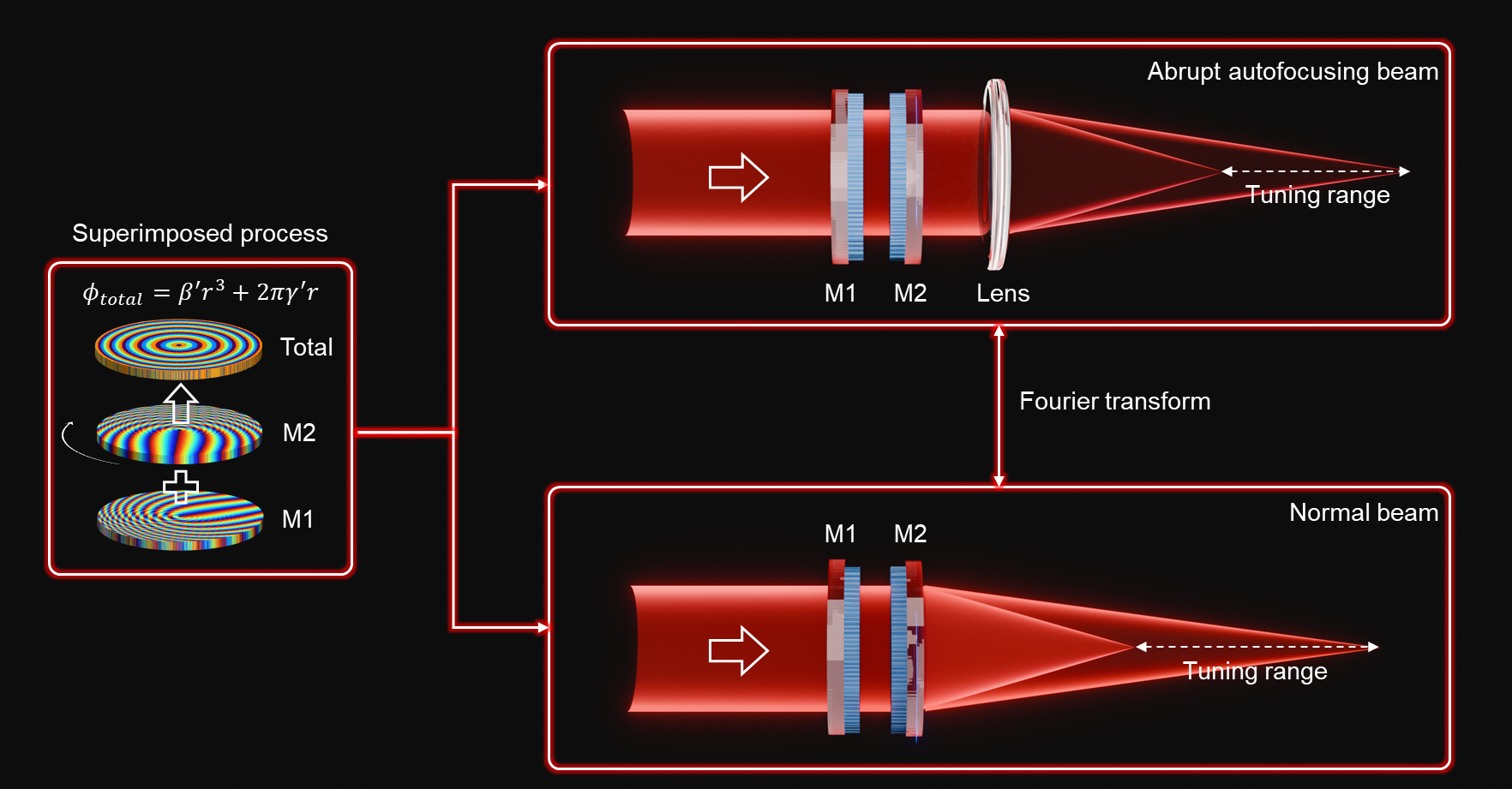


**Figure S1** Schematic illustration of the dual-mode beam generation enabled by a cascaded metasurface pair (M1 and M2). The total phase profile is synthesized as $\phi_{total}$, combining a cubic and a linear radial phase.The dual functionality arises from the same phase profile.

**Section 2: Refractive index of Si**


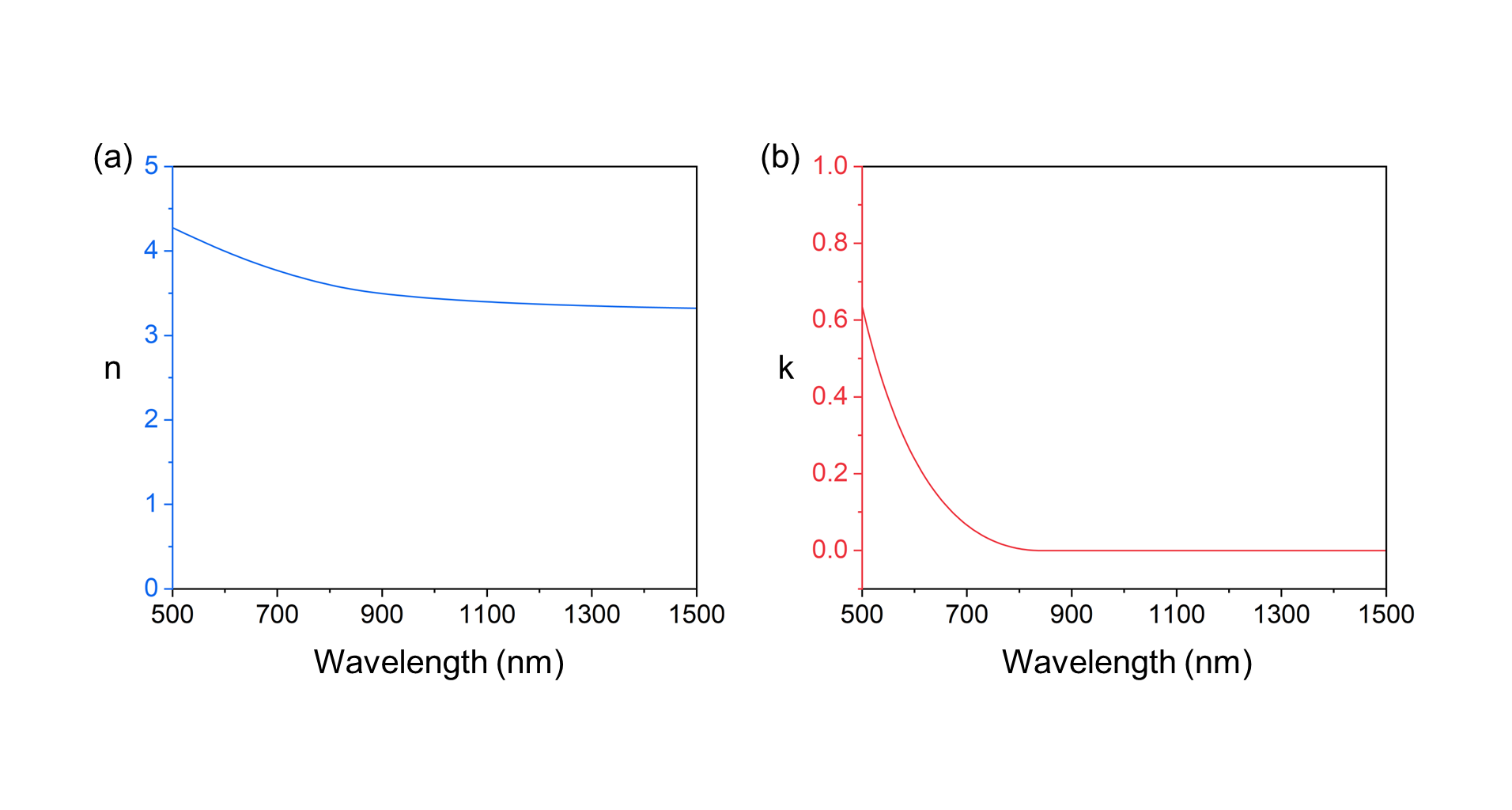


**Figure S2.** (a) Real part and (b) Imaginary part of the refractive index of Si film measured through an ellipsometer.

Spectroscopic ellipsometry was employed to characterize the refractive index of the Si film. The results reveal that the imaginary component remains negligible beyond 900 nm, indicating near-zero loss. Meanwhile, the real part of the refractive index exhibits minimal variation across the near-infrared region.

**Section 3: Optimization process of the applied metasurfaces**

In practical implementations, a finite air gap must be introduced between the two cascaded metasurfaces to prevent physical damage to the fabricated nanostructures. However, this gap allows the output field from the first metasurface (M1) to propagate freely before reaching the second (M2), resulting in a deviation of the actual superimposed phase distribution from the predesigned target. This mismatch degrades the quality of the generated optical beam, particularly in applications requiring high phase fidelity.

To evaluate the impact of interlayer spacing, we performed simulations using four different gap values: 0.2 mm, 0.5 mm, 1 mm, and 2 mm. The resulting focal plane intensity distributions were quantitatively compared to the ideal case (i.e., 0 mm gap) using three metrics: Mean Squared Error (MSE), MSE Percentage, and Structural Similarity Index (SSIM). Detailed definitions of these metrics are provided in Table S1. As shown in Figure S3, increasing the interlayer gap leads to higher MSE and MSE%, and a decrease in SSIM. However, the performance degradation introduced by a 1 mm gap remains minor. For instance, across different rotation angles, the MSE% at a 1 mm gap remains below 0.2%, and the SSIM stays around 0.8. These results suggest that the deviation introduced by a 1 mm gap is relatively small and can be considered acceptable in practical implementations.

**Table S1** Summary of evaluation Metrics

**
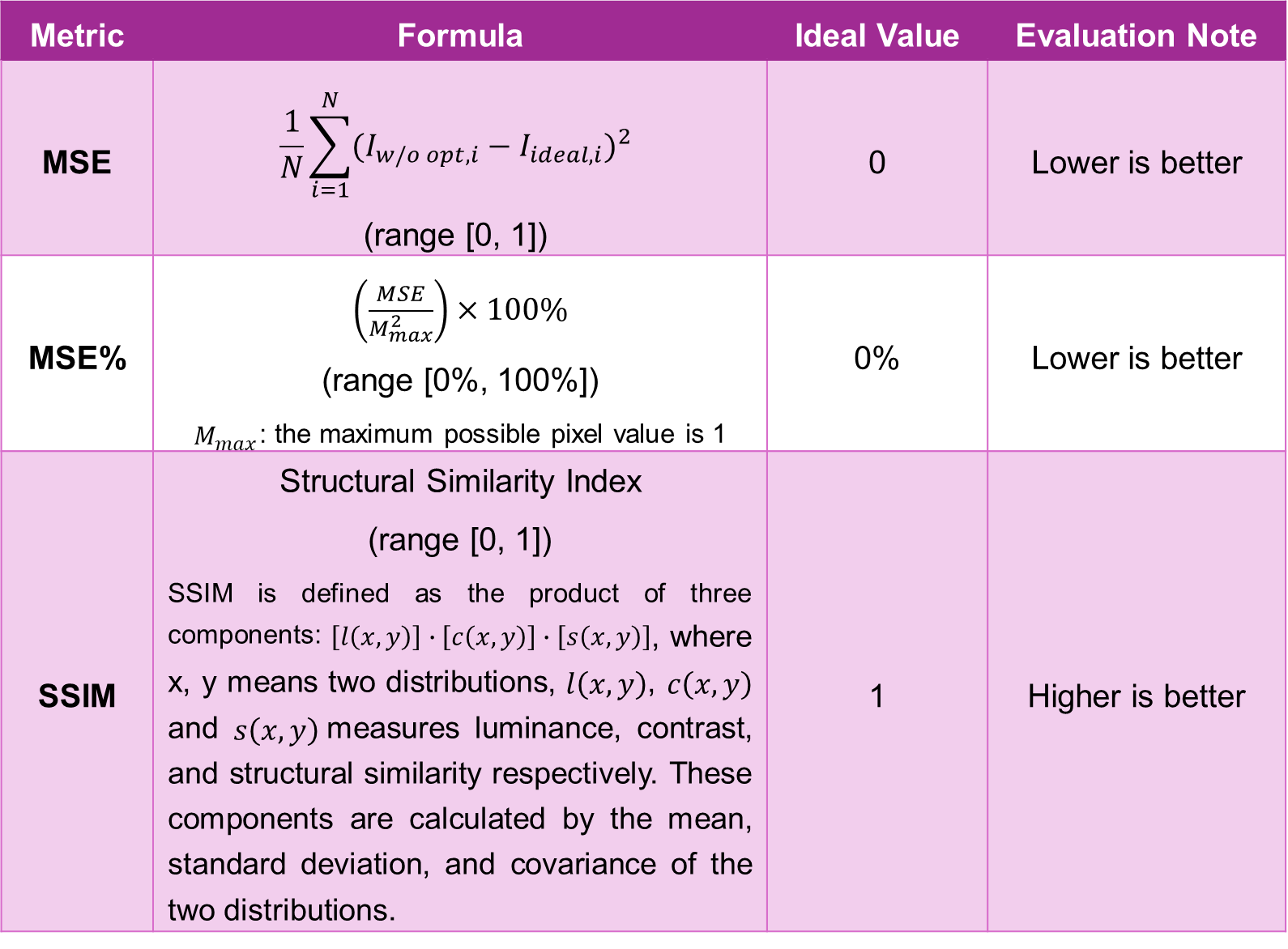
**

**
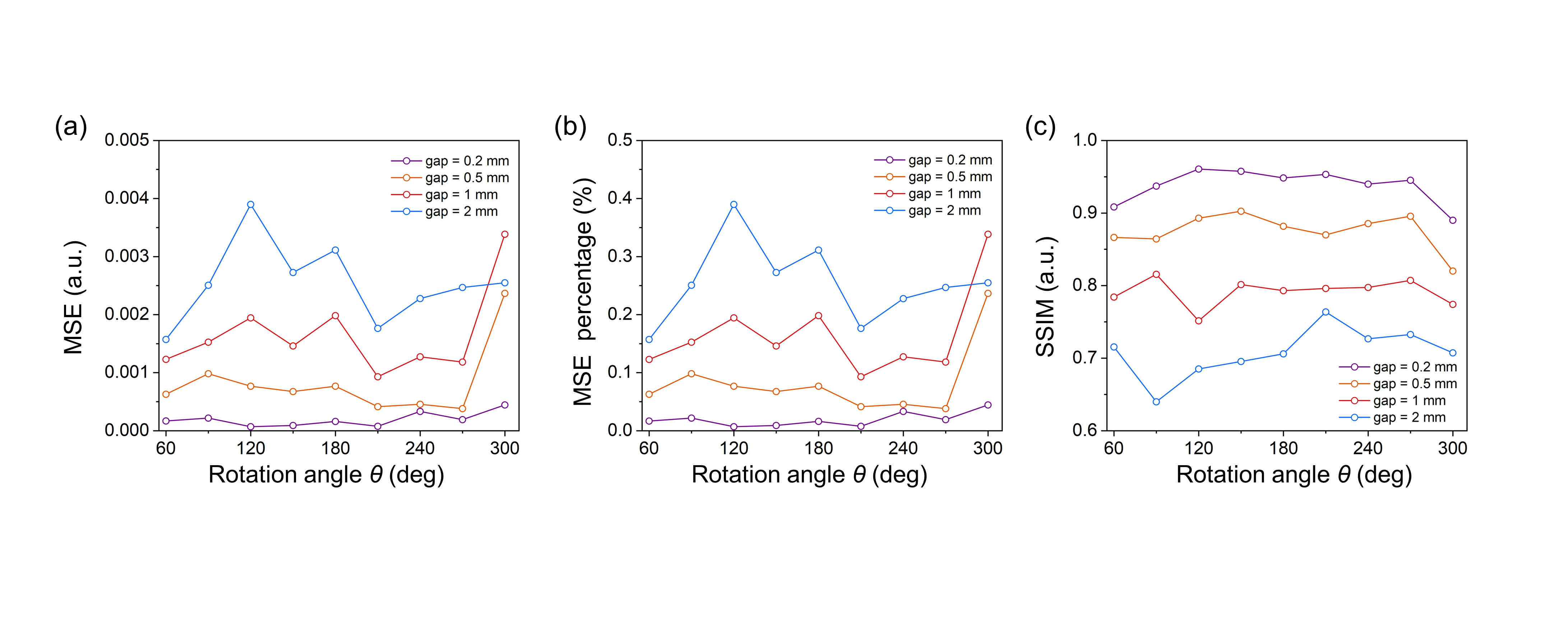
**

**Figure S3**. Quantitative evaluation of the focal plane intensity distributions under varying interlayer spacings between cascaded metasurfaces. (a) Mean squared error (MSE), (b) MSE percentage, and (c) structural similarity index (SSIM) between the resulting focal plane and the ideal case, as functions of the rotation angle 𝜃. Four gap values are compared: 0.2 mm, 0.5 mm, 1 mm, and 2 mm.

To mitigate this deviation effect while preserving structural integrity, we introduce an optimization strategy based on the Rayleigh–Sommerfeld diffraction framework. Specifically, we back-propagate the ideal phase distribution of M1 (Figure S4(a), left panel) by 1 mm which is the designated interlayer spacing, to retrieve a modified phase profile that compensates for free-space propagation. This optimized phase distribution (Figure S4(a), middle panel) is then used to fabricate M1, ensuring that the resultant superimposed phase after M2 (Figure S4(a), right panel) closely matches the intended design.

To quantitatively assess the effectiveness of this approach, we compare three scenarios described in Figure S4(b): (left panel) the ideal case, where perfect phase profiles are assumed for M1 and M2 with no gap; (middle panel) the unoptimized case (‘w/o opt.’), where a 1 mm gap is introduced without phase correction; and (right panel) the optimized case (‘opt.’), where the corrected M1 phase is used alongside the ideal M2 under the same 1 mm gap condition. As illustrated in Figures S4(c–e), this optimization significantly improves the fidelity of the reconstructed intensity distribution for the 1 mm gap case. Specifically, the MSE% is reduced to approximately 0.1%, and the average SSIM increases accordingly. This demonstrates that the optimized phase design can effectively compensate for the non-zero interlayer spacing, bringing the performance close to the ideal configuration.

**
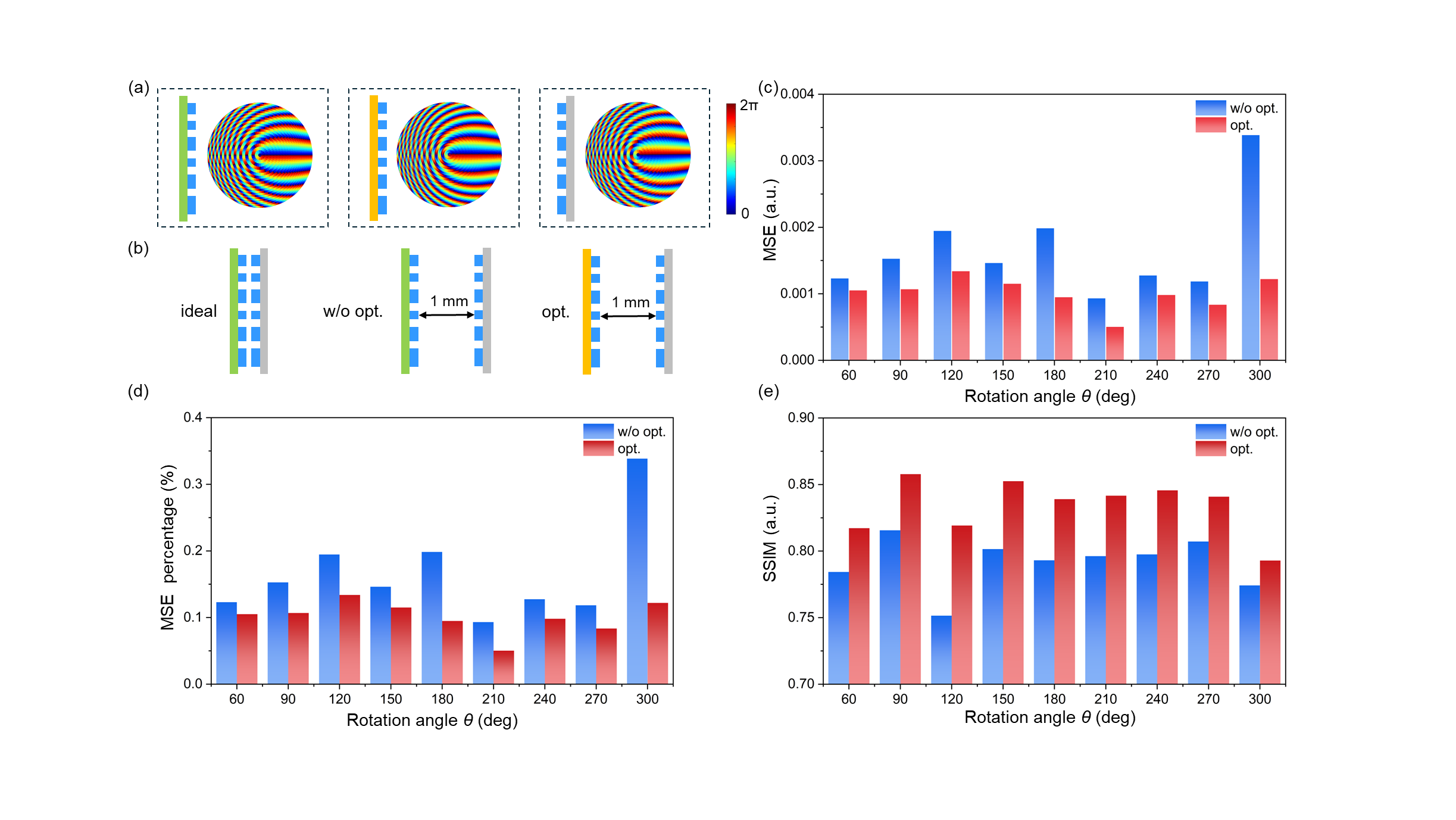
**

**Figure S4.** Optimization strategy to compensate for the effect of finite interlayer spacing. (a) Phase profiles of the metasurfaces: predesigned phase for M1 (left), optimized phase for M1 after Rayleigh–Sommerfeld-based back-propagation compensation (middle), and predesigned phase for M2 (right). (b) Schematic illustration of three system configurations: (ideal) metasurfaces in direct contact (0 mm gap) with predesigned phase profiles; (w/o opt.) 1 mm interlayer gap with uncorrected predesigned phases; and (opt.) 1 mm gap with optimized M1 phase and predesigned M2 phase. (c–e) Quantitative comparison of focal plane intensity distributions between the optimized and unoptimized cases across different rotation angles: (c) MSE, (d) MSE percentage, and (e) SSIM.

We also simulated the light intensity distribution in the x–z plane and extracted the focal length and FWHM of the standard focused beam under different gap conditions. The rotation angle was fixed at 120°, and the phase distribution has not been optimized. As shown in Figure S5, the focusing behavior remains stable and consistent for gap values up to 1 mm. The 2 mm configuration results in noticeable aberrations and reduced focusing quality, further supporting our selection of 1 mm as an optimal and robust spacing in both optical and mechanical terms.


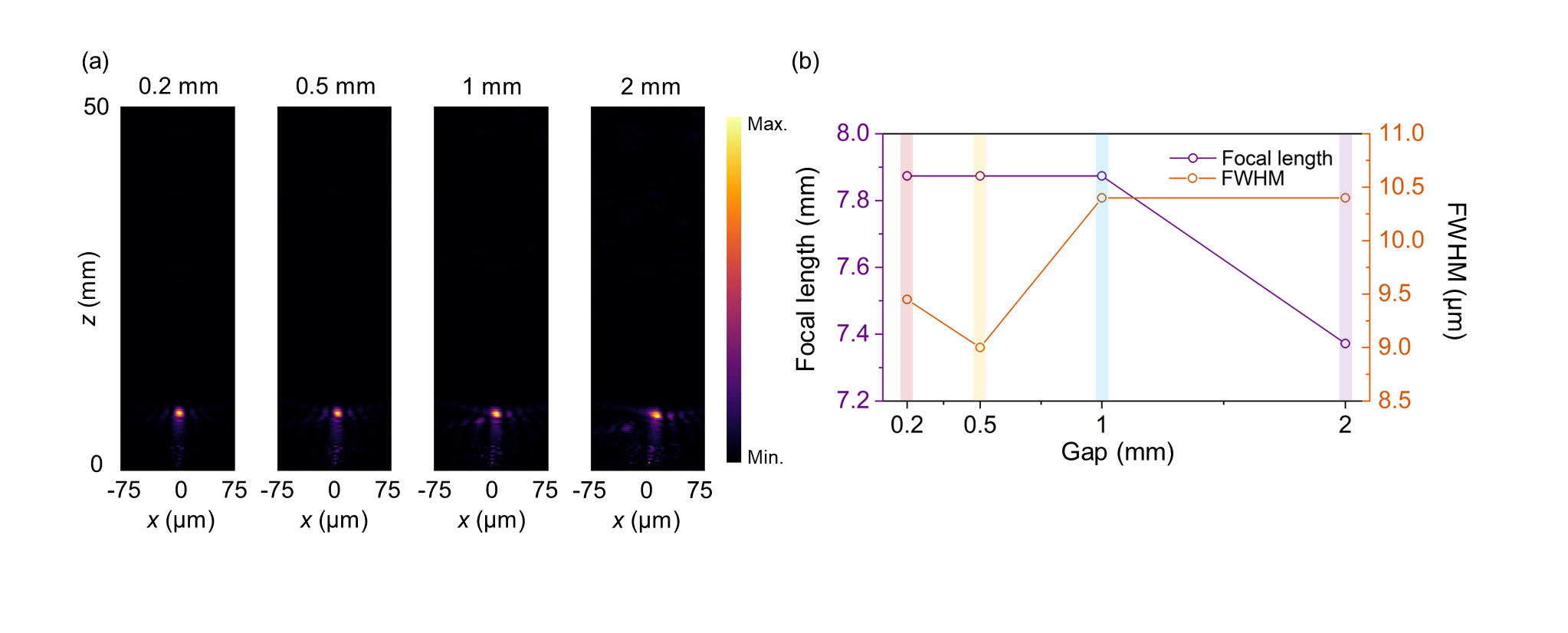


**Figure S5**. (a) x-z intensity distributions at different gaps, rotation angles are set as 120°. (b) Corresponding Focal length and FWHM of focal spot at different gaps.

It should be emphasized that the 1 mm spacing used in this work is suitable for our experimental conditions but not a strict requirement. With the proposed optimization strategy, the phase profile can be readily adapted for different spacing values. If the experimental setup works for a shorter wavelength, selecting a reduced distance would further help preserve the beam quality and improve overall performance.

The influence of angular misalignment (i.e., non-parallelism) between the two metasurfaces has been systematically investigated. As shown in Figure S6(a), we consider three representative configurations: (ideal) contact-aligned metasurfaces (0 mm gap) with predesigned phase profiles; (w/o opt.) a 1 mm interlayer gap with uncorrected predesigned profiles; and (opt.) a 1 mm gap with an optimized M1 phase, while M2 experiences an out-of-plane rotation angle θₚ. Figure S6(b) presents the transmittance and propagation phase of the unit cell as functions of the structural parameter D at an incident wavelength of 1310 nm under different θₚ values (0°, 5°, and 10°). These results indicate that slight out-of-plane rotations have minimal impact on the optical response of individual meta-atoms. Following the same evaluation methodology as in the interlayer spacing analysis, Figures S6(c) and S6(d) report the MSE, MSE percentage, and SSIM values of focal plane intensity distributions between the optimized and unoptimized cases as functions of the rotation angle θ for θₚ = 5° and 10°, respectively. The optimized condition consistently yields lower MSE/MSE% and higher SSIM. Additionally, Figure S6(e) shows the intensity distributions under the optimized condition for different θₚ at θ = 120°. These results demonstrate that the proposed method maintains high performance even when the two metasurfaces are not perfectly parallel.


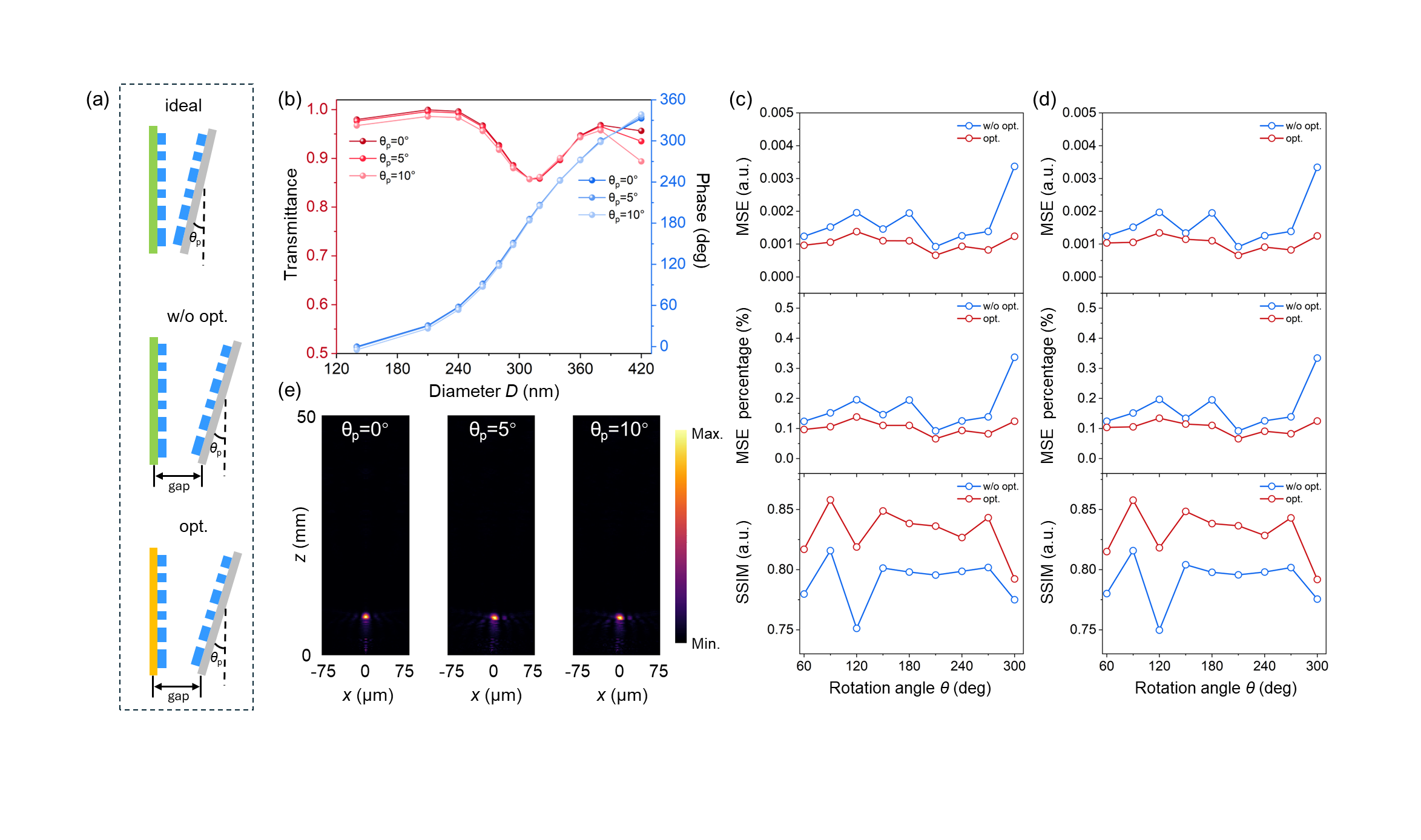


**Figure S6.** (a) Schematic representation of three system configurations: (ideal) contact-aligned metasurfaces (0 mm gap) with predesigned phase profiles; (w/o opt.) 1 mm interlayer gap with uncorrected predesigned profiles; (opt.) 1 mm gap with optimized M1 phase and predesigned M2. In all configuration, M2 undergoes an out-of-plane rotation with angle θ_p. (b) Transmittance and propagation phase of the unit cell as functions of diameter D at an incident wavelength of 1310 nm under different θ_p. (c,d) MSE, MSE percentage, and SSIM of focal plane intensity distributions between the optimized and unoptimized cases as functions of the rotation angle 𝜃, corresponding to θ_p=5^°and 10^°respectively. (e) Intensity distribution under the optimized condition for standard focusing mode.

The influence of lateral alignment error has also been investigated. As illustrated in Figure S7(a), we consider three configurations: (ideal) contact-aligned metasurfaces (0 mm gap) with lateral shift d; (w/o opt.) 1 mm interlayer distance with a lateral displacement d between the two layers and no optimization; and (opt.) 1 mm distance with misalignment d but with an optimized phase profile for M1. The lateral displacement d is defined as a fraction of the aperture size D, and we study two representative cases where d = 1/100 D and d = 1/50 D. We quantitatively analyze the focal spot intensity distributions performance via MSE, MSE percentage, and SSIM metrics as functions of rotation angle θ, as summarized in Figures S7(b) and S7(c) corresponded to d = 1/100 D and d = 1/50 D. Across different angles, the optimized condition consistently achieves lower MSE and MSE%, and higher SSIM, validating the robustness of the optimization strategy against lateral misalignments. In addition, The resulting intensity distributions under the optimized condition at θ=120^° are shown in Figure S7(d). It exists a slightly lateral displacement while achieving standard focusing function. These results confirm that the proposed optimization framework is widely useful not only to interlayer distance and parallelism but also to practical lateral positioning errors.


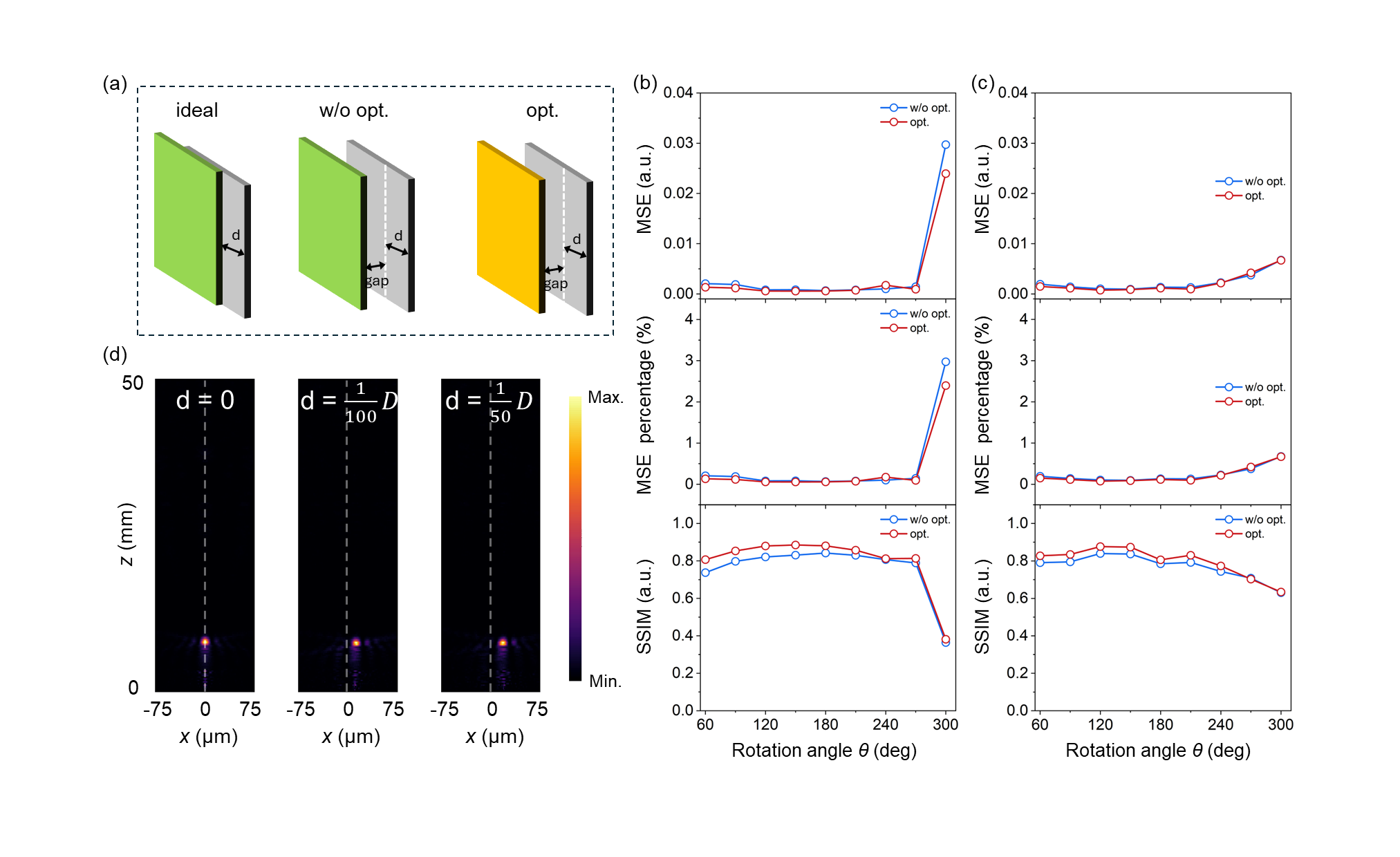


**Figure S7.** (a) Schematic illustration of three system configurations: (ideal) contact-aligned metasurfaces with lateral shift d; (w/o opt.) 1 mm interlayer gap and lateral shift d without optimization; (opt.) 1 mm gap with lateral misalignment d and optimized M1 phase. (b, c) MSE, MSE percentage, and SSIM as functions of rotation angle θ for d = 1/100 D and d = 1/50 D, respectively. (d) Simulated intensity distributions at d = 0, 1/100 D, and 1/50 D under the optimized condition, demonstrating the robustness of the proposed method.

**Section 4: SEM images of the fabricated metasurfaces**

**
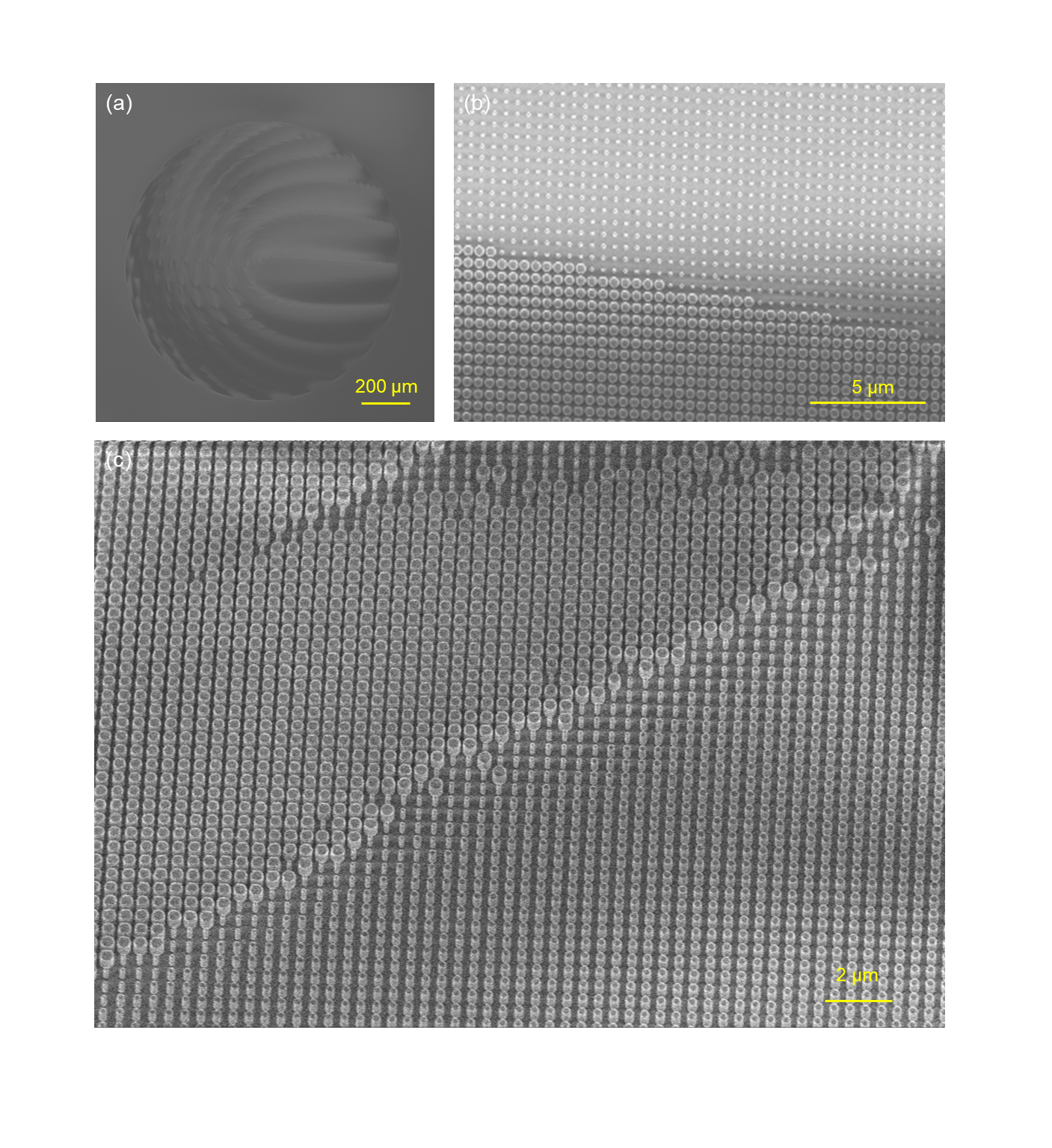
**

**Figure S8.** SEM images with different magnifications. (a) M2, scale bar: 200 μm. (b) M1, scale bar: 5 μm. (c) M2, scale bar: 2 μm.

**Section 5: SEM images of the fabricated metasurfaces**


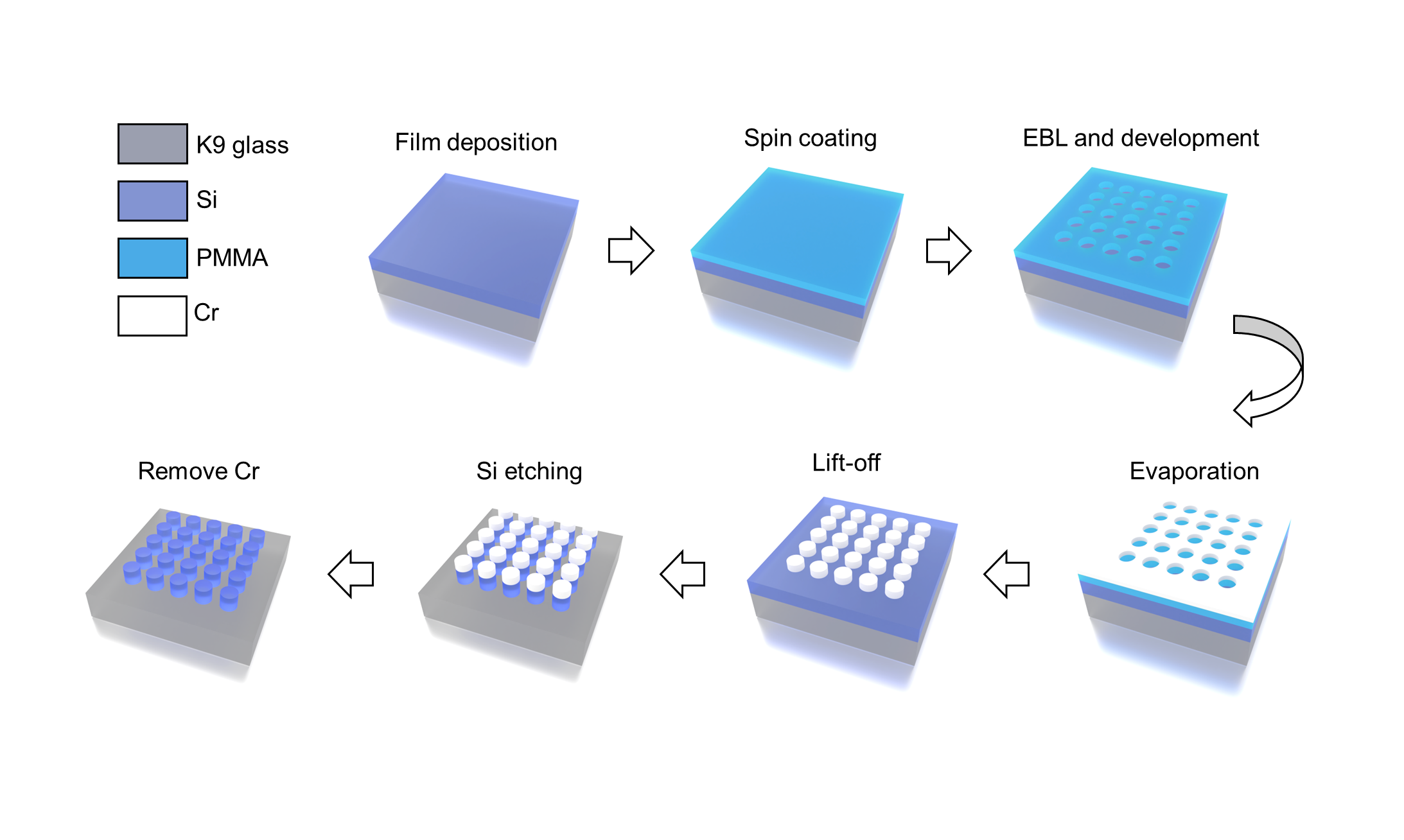


**Figure S9.** Fabrication process of Si metasurfaces.

**Section 6: Numerical analysis for generating tunable AAF beams**

In the numerical analysis, the Rayleigh–Sommerfeld diffraction algorithm was employed to simulate the optical field distribution generated by the varifocal meta-lens. To balance computational efficiency with accuracy, the Fourier lens used in the simulation was set to be three times larger in aperture than the meta-lens, ensuring complete collection of the transmitted light. Figure S10(a) illustrates the computed *xz-*plane intensity distributions at various rotation angles, with a fixed numerical aperture (NA) of 0.26 for the Fourier lens.

Figures S10(b) and S10(c) display the transverse *xy-*plane intensity distributions at two key planes: the back focal plane of the Fourier lens (also referred to as the initial plane for AAF beam formation) and the focal plane of the resulting AAF beam, respectively. As shown in Figure S10(d), the focal length increases monotonically with the rotation angle *θ*, in agreement with experimental observations. While the focusing efficiency exhibits noticeable variation across rotation angles, the beam’s abrupt autofocusing behavior remains robust. Enhancing the overall focusing efficiency, especially under large-angle rotations, remains a relevant direction for future optimization.

Apart from focal length tuning, the beam profile at the initial plane also shows strong dependence on rotation angle *θ*. Specifically, the concentric circle pattern first expands and then contracts as θ increases. To quantify this behavior, we define the diameter *D_r_* of the main lobe (see inset of Figure S10(e)) as a metric for beam size.

Importantly, the full width at half maximum (FWHM) of the focal spot remains stable at approximately 4.5 μm over the entire tuning range, confirming the beam’s non-diffracting nature. Another characteristic of AAF beams—the high intensity contrast between the initial and focal planes—is also preserved. This feature is particularly advantageous for deep-tissue laser applications, where high focal intensity is desired without significant surface damage. Figure S10(f) shows that the intensity ratio remains above 100 for *θ* ≤ 210°, reaching a maximum value of 325 at *θ* = 120°.


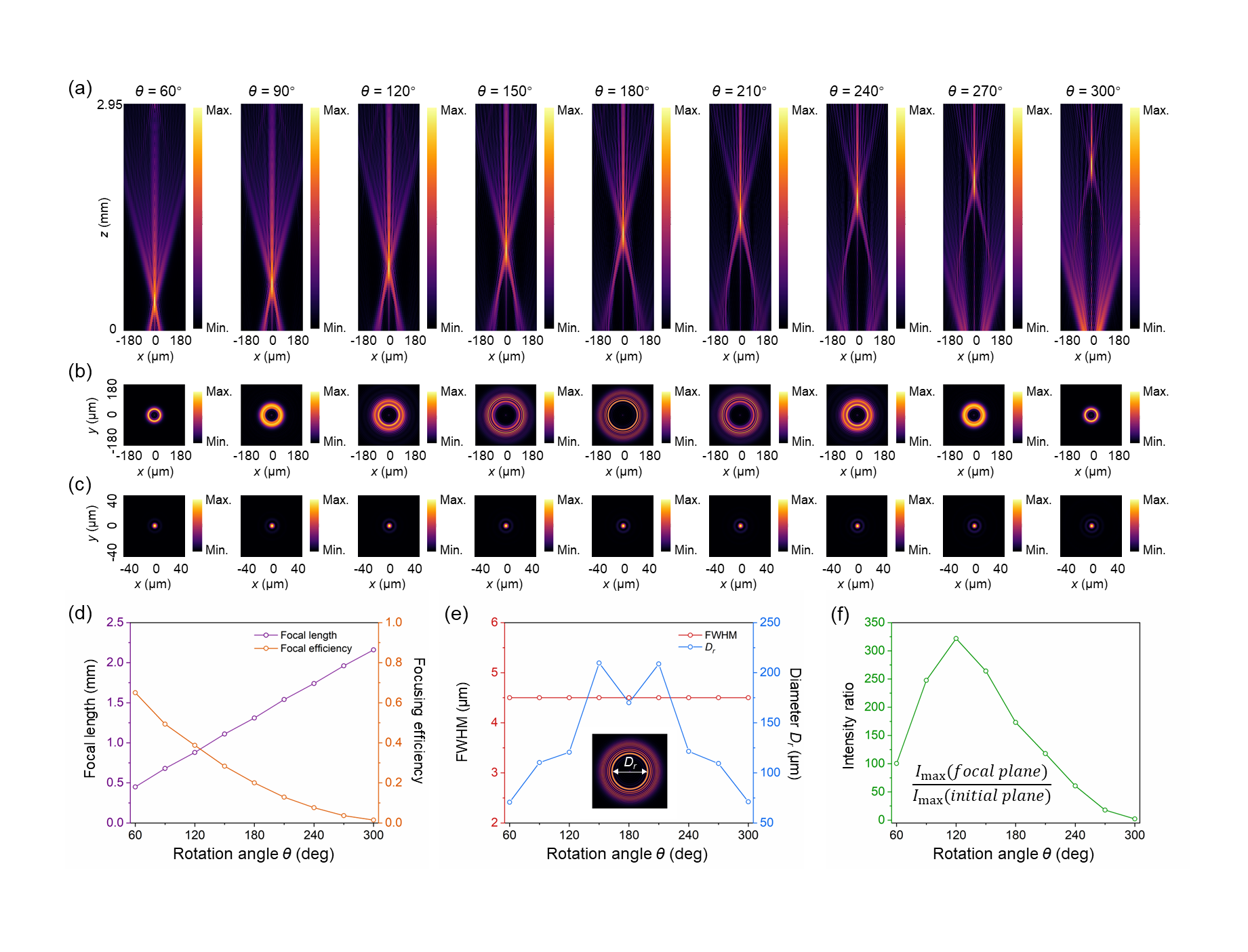


**Figure S10.** Numerical simulation of tunable AAF beam formation using a Fourier lens with NA = 0.26. (a) Simulated *xz*-plane intensity distributions at various relative rotation angles *θ*. (b) Corresponding *xy*-plane intensity distributions at the back focal plane of the Fourier lens (initial plane for AAF beam formation). (c) *xy*-plane intensity distributions at the focal plane of the generated AAF beams. (d) Extracted focal length and focusing efficiency as a function of *θ*. (e) FWHM of the focal spot and the main-lobe diameter *D_r_* of the concentric circle at the initial plane. (f) Intensity ratio between the focal plane and initial plane.

It is worth noting that the tunable AAF beam can be generated using Fourier lenses with different NA. Figure S11 presents the simulation results when the NA of the Fourier lens is increased to 0.42. As shown in Figures S11(a–c), the characteristic autofocusing behavior is preserved, confirming that the AAF beam formation is not limited to a specific NA value.

The overall trends—including focal length, focusing efficiency, diameter of the main lobe at the initial plane, FWHM of the focal spot, and intensity ratio—remain consistent with those observed under NA = 0.26. However, as expected, the absolute values of these parameters vary with the choice of Fourier lens, as illustrated in Figures S11(d–f).

This observation also explains the quantitative differences between experimental and simulated results: in practice, the Fourier lens used in the optical setup differs in NA and aperture from that adopted in the numerical simulation. Moreover, it should be emphasized that the Fourier lens used in the experimental setup is a commercial microscope objective rather than an ideal thin lens. Compared to conventional Fourier lenses, objectives exhibit more complex optical behavior due to their multi-element design, higher numerical aperture, and inherent aberration correction. These factors influence the beam’s propagation characteristics, leading to slight deviations in focal length, ring diameter, and spot size when compared to idealized numerical simulations. Such discrepancies are therefore expected and do not undermine the validity of the observed AAF beam formation.


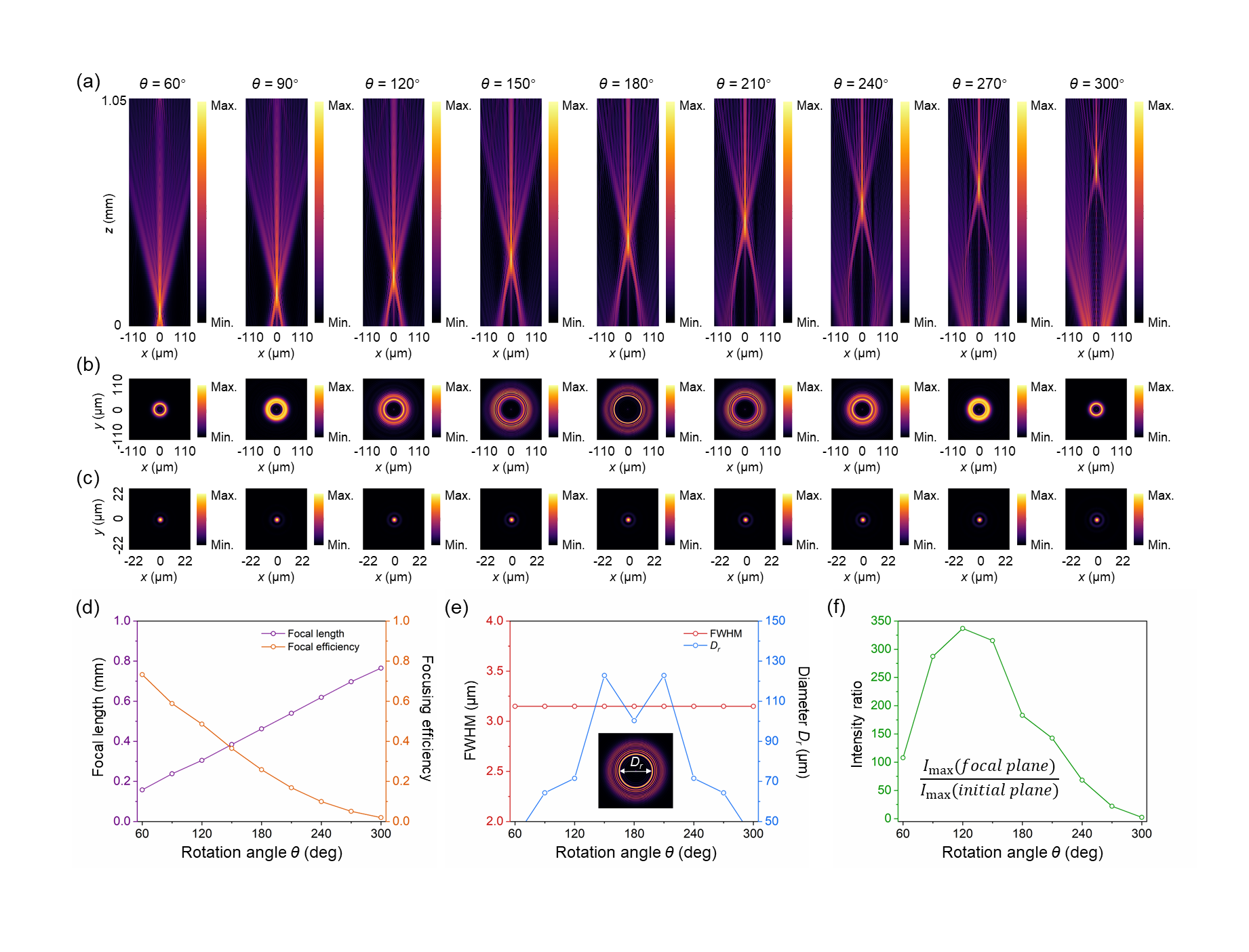


**Figure S11.** Numerical simulation of tunable AAF beam formation using a Fourier lens with NA = 0.42. (a) Simulated *xz*-plane intensity distributions at various relative rotation angles *θ*. (b) Corresponding *xy*-plane intensity distributions at the back focal plane of the Fourier lens (initial plane for AAF beam formation). (c) *xy*-plane intensity distributions at the focal plane of the generated AAF beams. (d) Extracted focal length and focusing efficiency as a function of *θ*. (e) FWHM of the focal spot and the main-lobe diameter *D_r_* of the concentric circle at the initial plane. (f) Intensity ratio between the focal plane and initial plane.

**Section 7: Experimental intensity distributions of the generated AAF beam**


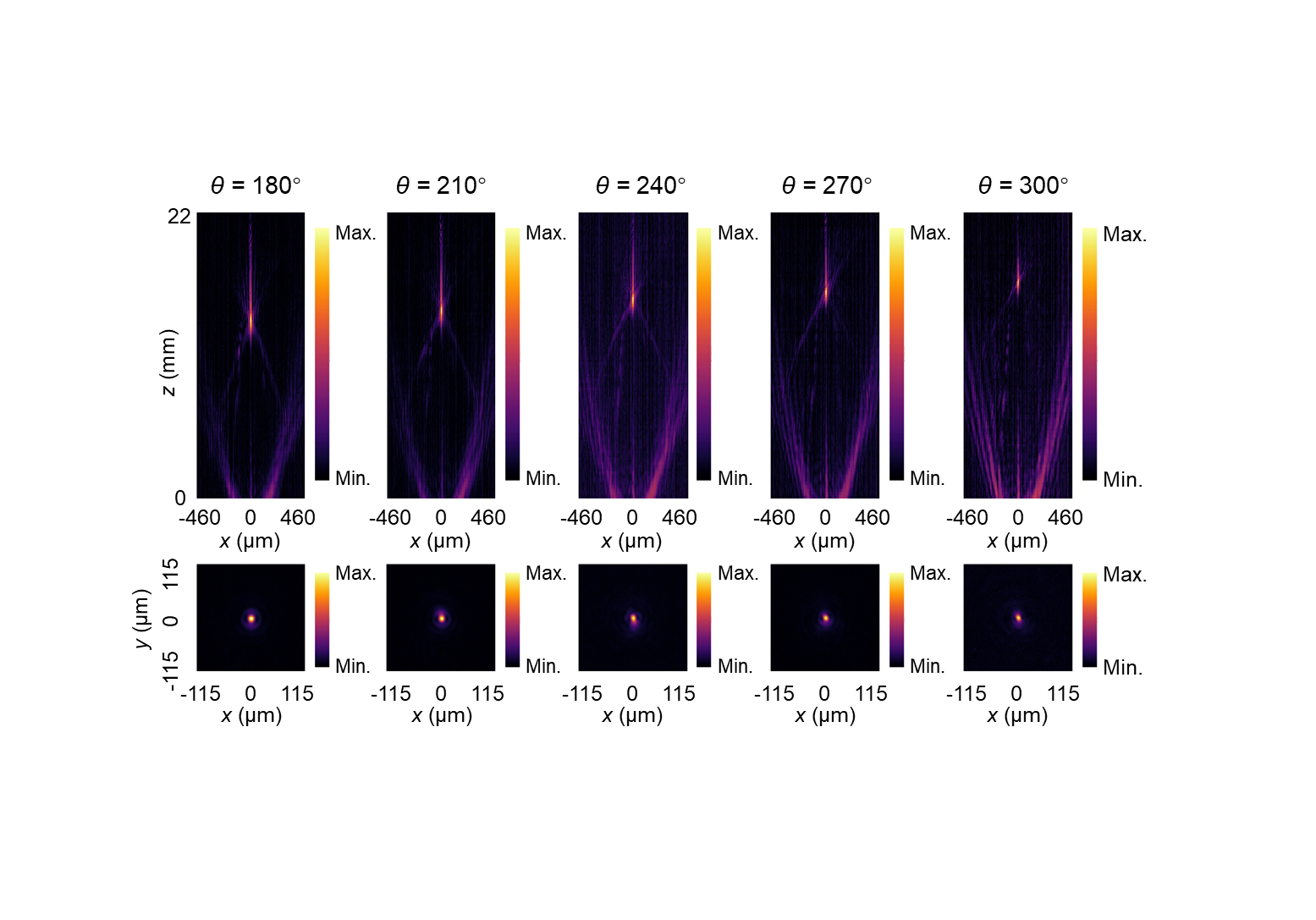


**Figure S12.** Experimental results of tunable AAF beam. Experimental *xz-*plane intensity cross-sections and corresponding *xy-*plane intensity distributions at the focal plane under varying relative rotation angles *θ* of 180°, 210°, 240°, 270°, and 300°.

**Section 8: Numerical analysis of tunable AAF beam with selective blocking**


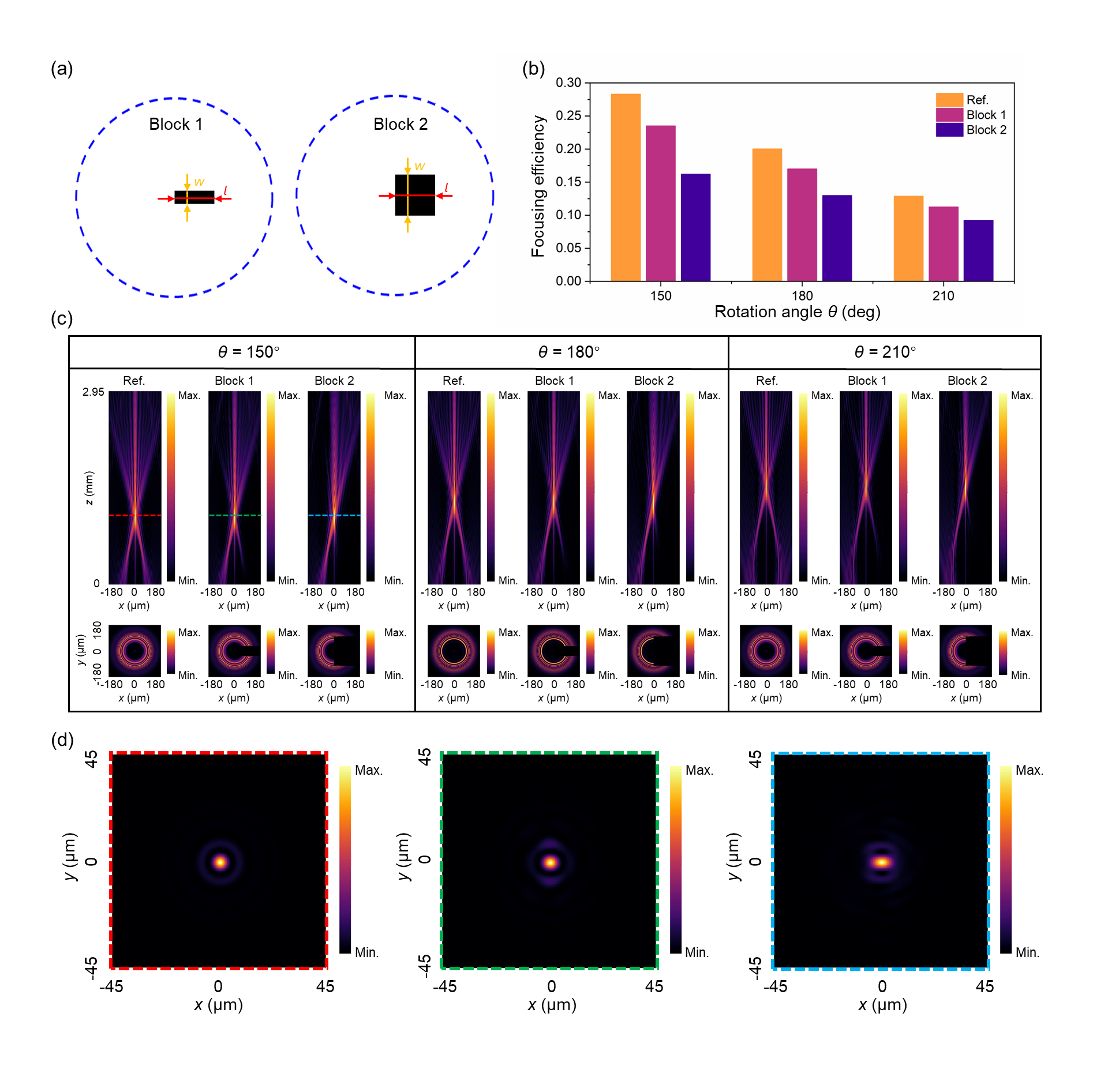


**Figure S13.** Numerical simulations of tunable AAF beam with selective blocking. (a) Description of two different blocks. Block 1: *w* = 67.5 µm, *l* = 202.5 µm; Block 2: *w* = 202.5 µm, *l* = 202.5 µm. Blue dashed line represents the diameter of meta-lens. (b) Focusing efficiencies under three conditions including unobstructed propagation, Block 1 obstruction, and Block 2 obstruction. (c) *x-z* intensity cross-sections and corresponding initial plane *x-y* intensity distributions at different rotation angles under three conditions. (d) Focal plane *x-y* intensity distributions at *θ* = 90°.

To evaluate the self-healing capability of the generated tunable AAF beam, two types of opaque obstacles were introduced at the initial plane. The dimensions and relative positions of the obstacles with respect to the varifocal metalens are illustrated in Figure S13(a), where the blue dashed circle indicates the aperture of the metalens. Block 1 has a width of *w* = 67.5 μm and length of *l* = 202.5 μm, while Block 2 is larger, with *w* = 202.5 μm and *l* = 202 μm.

We investigated the impact of these obstructions on the beam’s focusing performance at three representative relative rotation angles: 150°, 180°, and 210°. As shown in Figure S13(b), increasing the size of the opaque obstacle leads to a moderate reduction in focusing efficiency. However, the autofocusing functionality of the AAF beam remains intact, indicating strong robustness to partial beam blockage.

Figure S13(c) presents the simulated *x-z* intensity distributions and at the *x-y* initial plane under different obstruction conditions. While the beam maintains its overall propagation behavior, some degradation in the circular symmetry of the focal spot is observed, primarily due to asymmetric diffraction introduced during propagation. Figure S13(d) shows the focal-plane intensity profiles at *θ* = 150° for the three cases.

**Section 9: Experimental intensity distributions of the standard focusing beam**

**
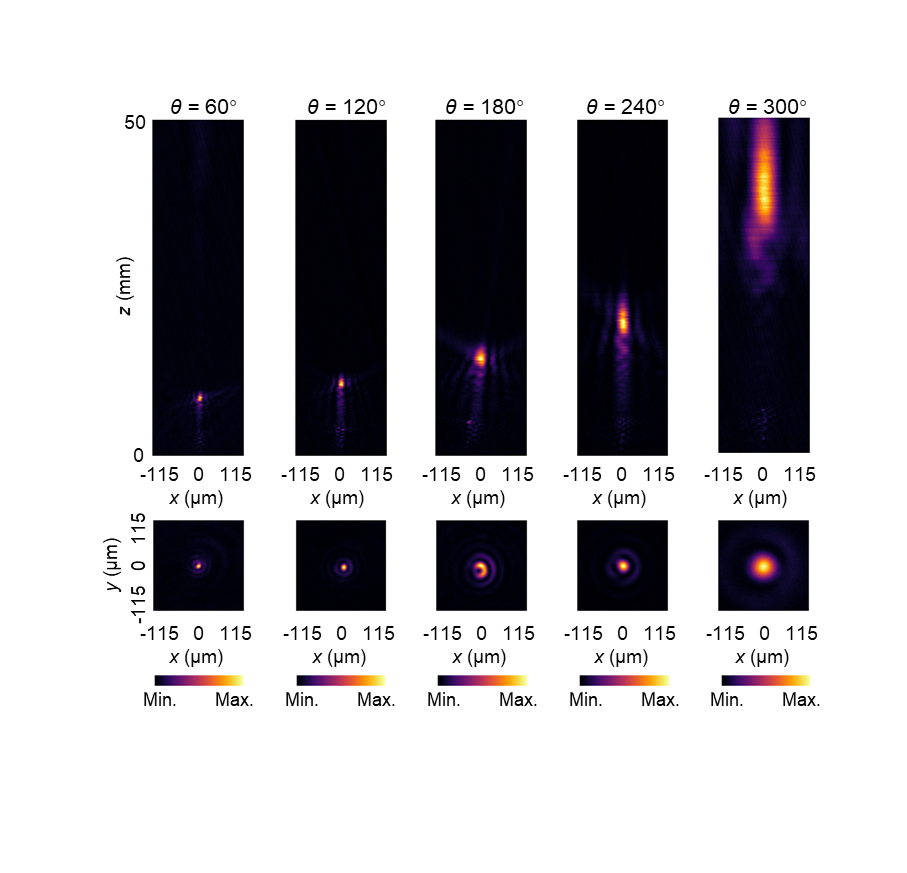
**

**Figure S14.** Experimental results of a tunable focusing beam. Experimental *x-z* intensity cross-sections and corresponding *x-y* intensity distributions at the focal plane under varying *θ* of 60°, 120°, 180°, 240°, and 300°.

As shown in Figure S14, the focal spot at *θ* = 180°exhibits noticeable degradation in symmetry and intensity uniformity, which can be attributed to imperfect spatial overlap of the optical phases from the two metasurfaces. Such misalignment disrupts the intended superposition of phase fronts, leading to beam distortion and reduced focusing performance.

To quantitatively assess the imaging performance under different rotation angles, Figure S15 shows three key metrics at different rotation angles: focusing efficiency, Strehl ratio, and modulation transfer function (MTF). The focusing efficiency increases with rotation angle, indicating improved energy concentration, and Strehl ratio also shows a rising trend with rotation angle. MTF was obtained from the Fourier transform of the PSF. While low-frequency components remain relatively stable, the transmission of high-frequency components decreases significantly at higher angles, especially at 𝜃=270°, due to main lobe broadening.

**
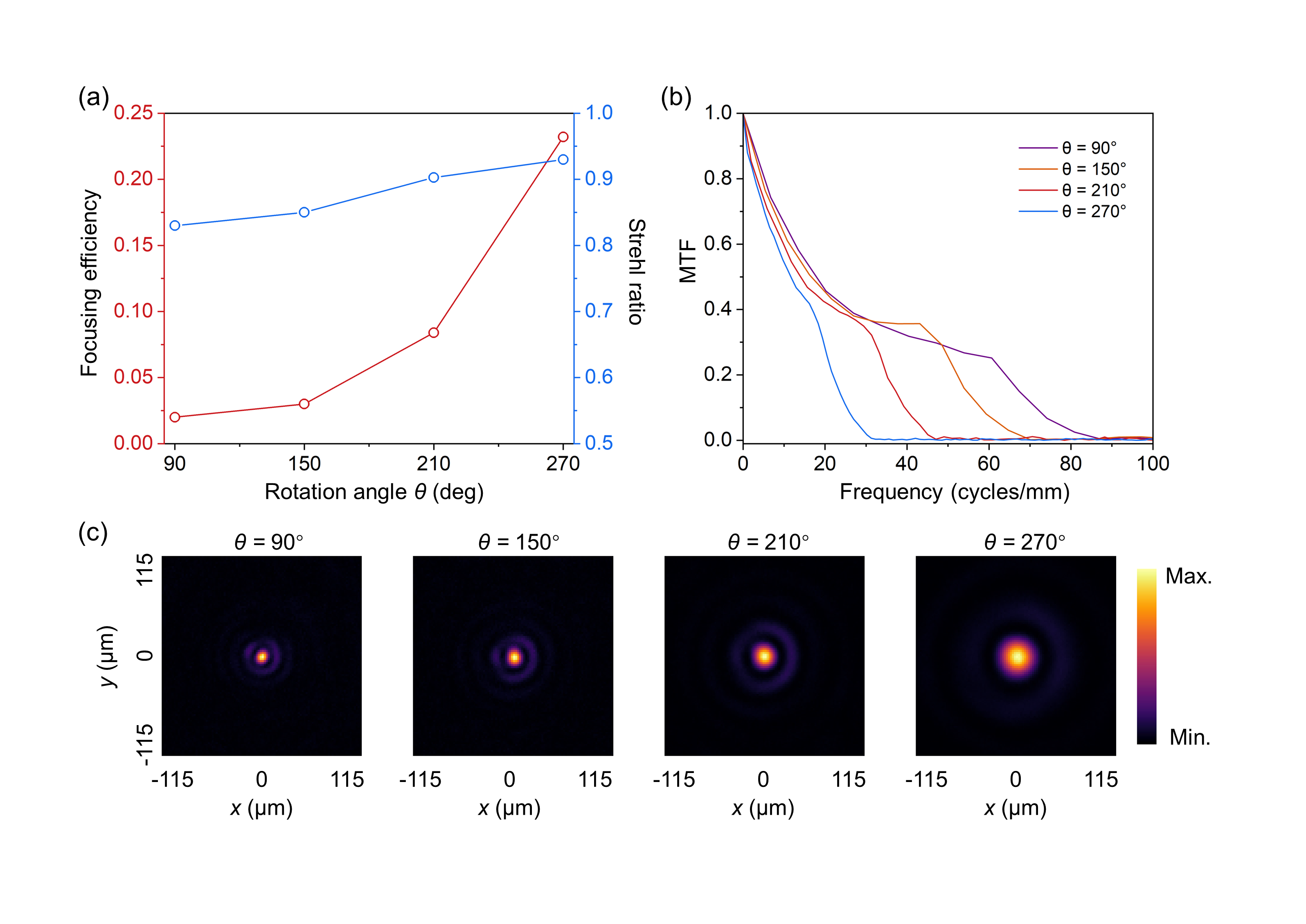
**

**Figure S15.** (a) Focusing efficiency and Strehl ratio as functions of rotation angle. (b) MTFs corresponding to four representative angles. (c) Experimentally measured PSF intensity distributions at different rotation angles.

**Section 10: Numerical analysis for standard focusing**


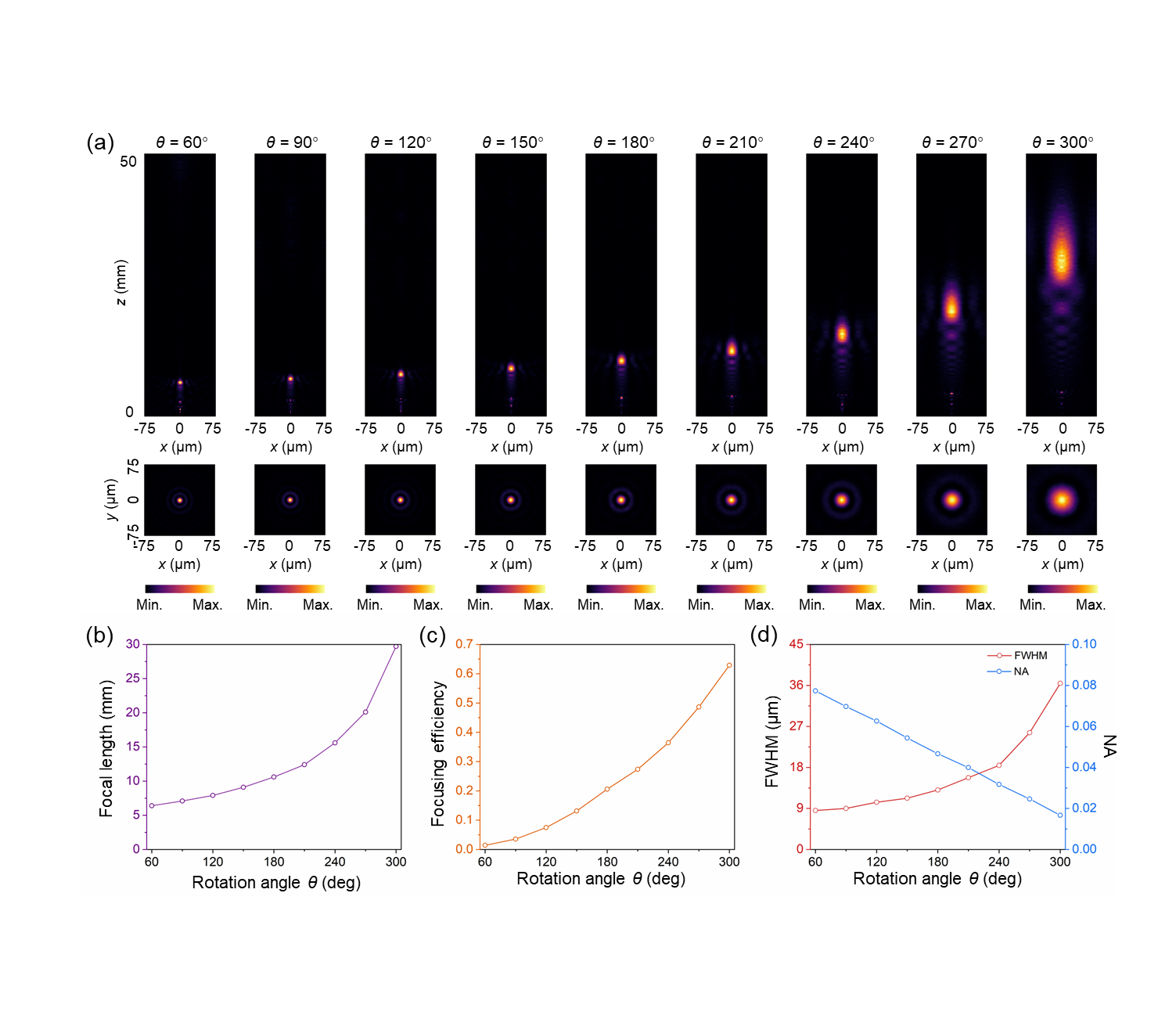


**Figure S16.** Simulated results of tunable focused beam. (a) *x-z* intensity distributions and *x-y* intensity distributions at different *θ*. (b) Focal length at different rotation angles. (c) Focusing efficiency at different *θ*. (d) FWHM of focal spot and corresponding NA at different *θ*.

Figure S16(a) presents the simulated intensity distributions of the standard focusing mode across different rotation angles. As the rotation angle increases, both the focal length and the FWHM of the focal spot exhibit a monotonic increase, which is consistent with experimental observations. The detailed focal length tuning range is summarized in Figure S16(b).

Figures S16(c) and S16(d) further illustrate the variation of focusing efficiency and FWHM of focal spot as functions of the rotation angle. Additionally, the effective NA corresponding to each rotation state was derived. These results confirm that even in the absence of abrupt autofocusing, the system still exhibits tunable focal characteristics governed by geometric rotation.

**Section 11: Comparison between our work and other representative varifocal meta-lenses**

**Table S2.** Comparison between our work and other representative varifocal meta-lenses

**
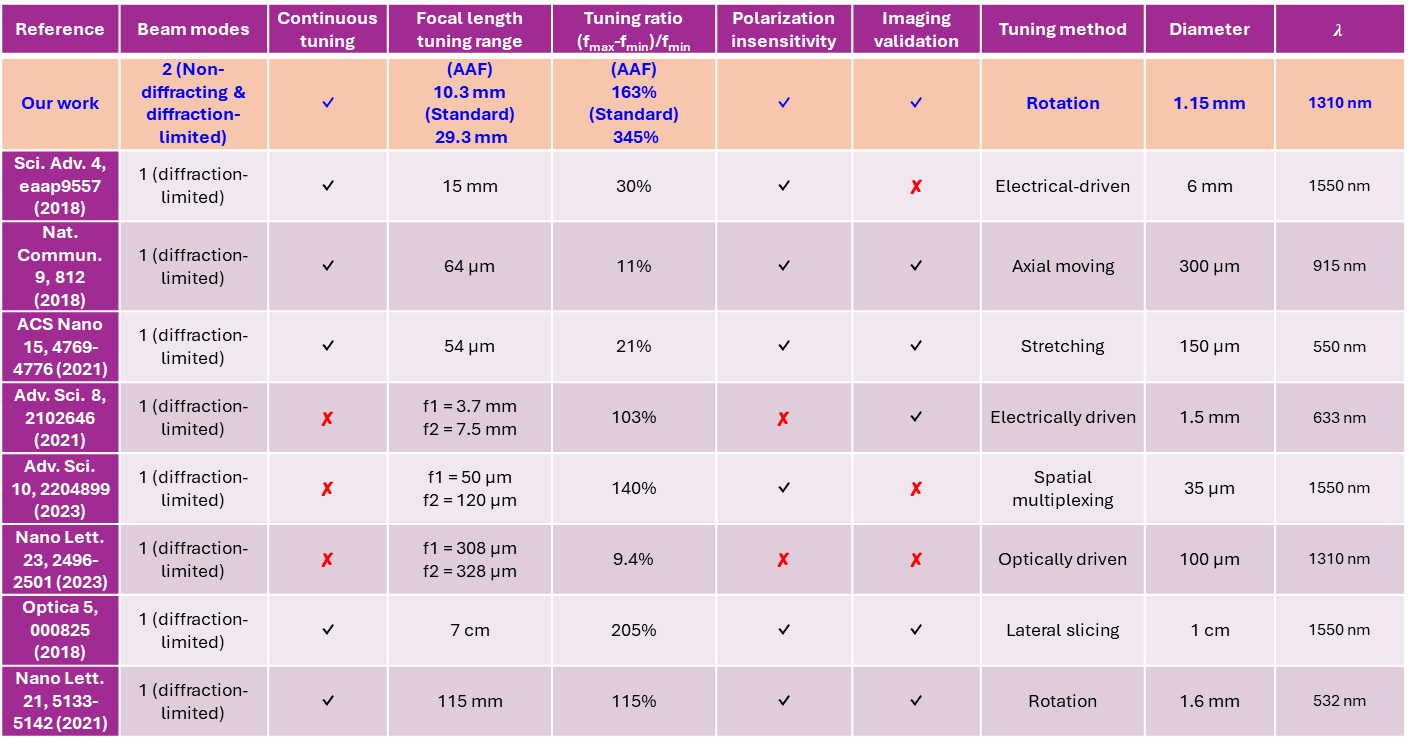
**

**References**

[1] D. G. Papazoglou, N. K. Efremidis, D. N. Christodoulides, S. Tzortzakis, *Optics Letters* **2011**, 36, 1842.

[2] J. A. Davis, D. M. Cottrell, D. Sand, *Opt Express* **2012**, 20, 13302.

[3] I. Chremmos, P. Zhang, J. Prakash, N. K. Efremidis, D. N. Christodoulides, Z. Chen, *Optics Letters* **2011**, 36, 3675.
